# Supplementary material for: The human host response to monkeypox infection: a proteomic case series study
Source: EMBO Mol Med. 2022 Sep 28;14(11):e16643. doi: 10.15252/emmm.202216643 (PMC9641420; doi:10.15252/emmm.202216643)
Supplement: Supplementary file 1 — Expanded View Figures PDF [file EMMM-14-e16643-s006.pdf]

Expanded View Figures

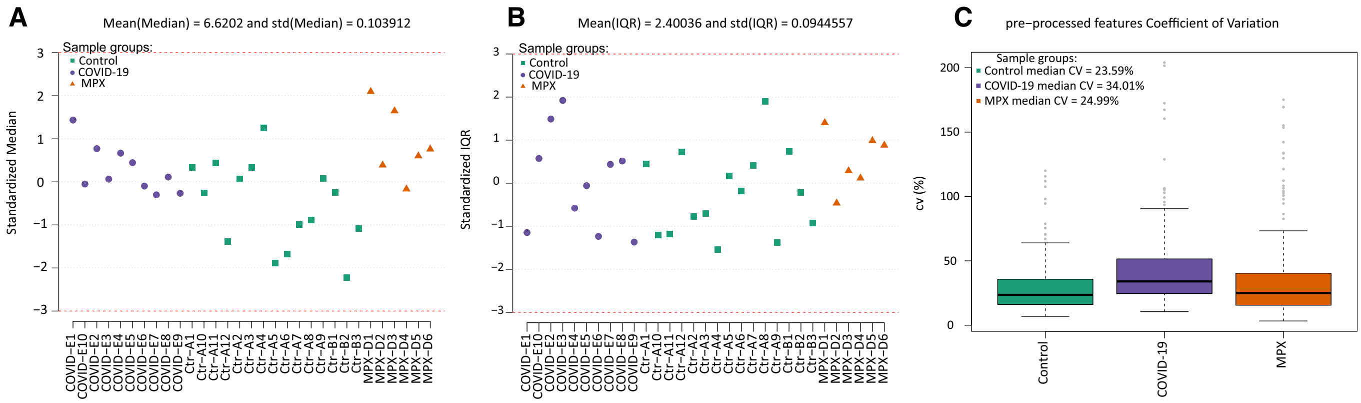

Figure EV1. Samples quality control (QC).

A QC chart of standardized median expression of proteins the study sample.

B QC chart of standardized interquartile range (IQR) of protein expressions in the study samples.

C Box plot of within group coefficient of variation of protein intensities.

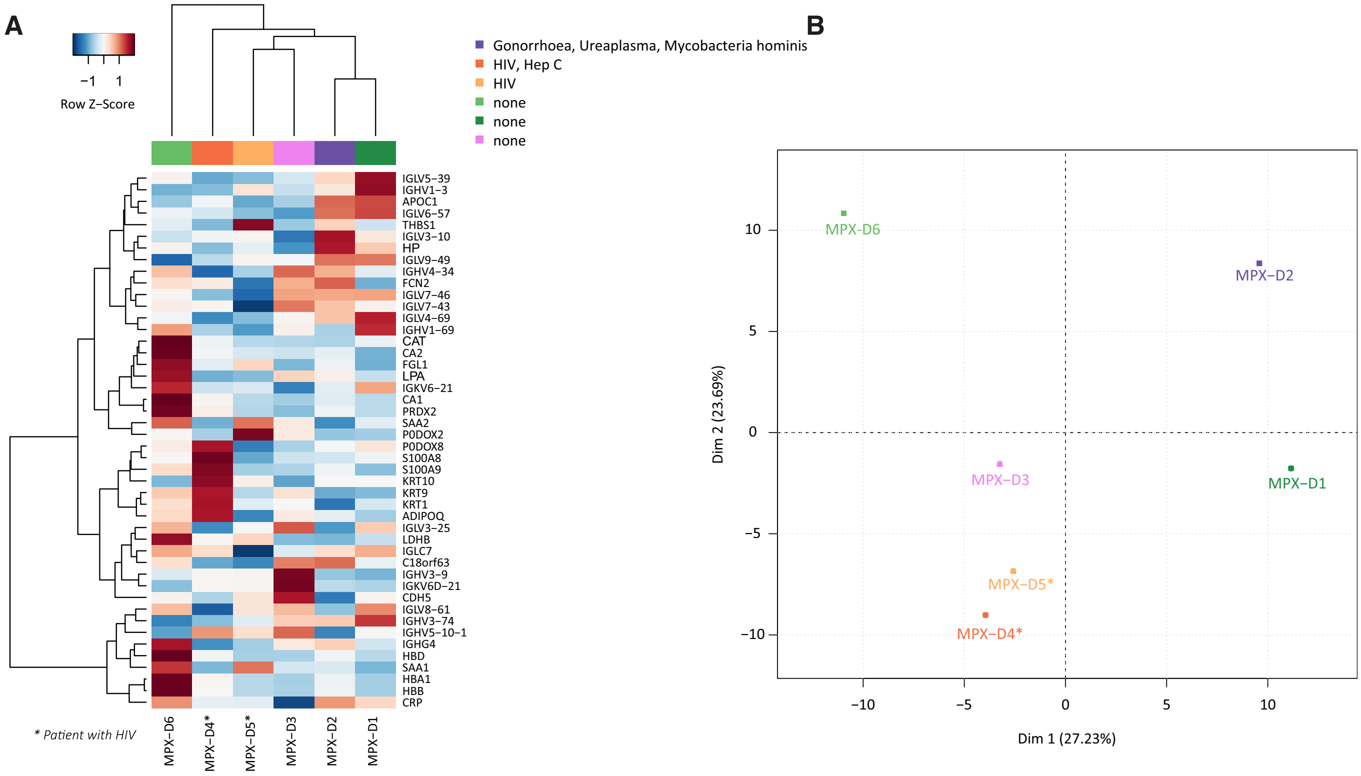

Figure EV2. Exploratory analysis of monkeypox samples clustering according to comorbidities.

A Hierarchical clustering using top 20% of most variable proteins. It is seen that HIV samples do not cluster together.

B PCA score plot using full proteome. It is seen that HIV is not a driving factor of samples variance. Its largest contribution is into the second principal component.

© 2022 The Authors

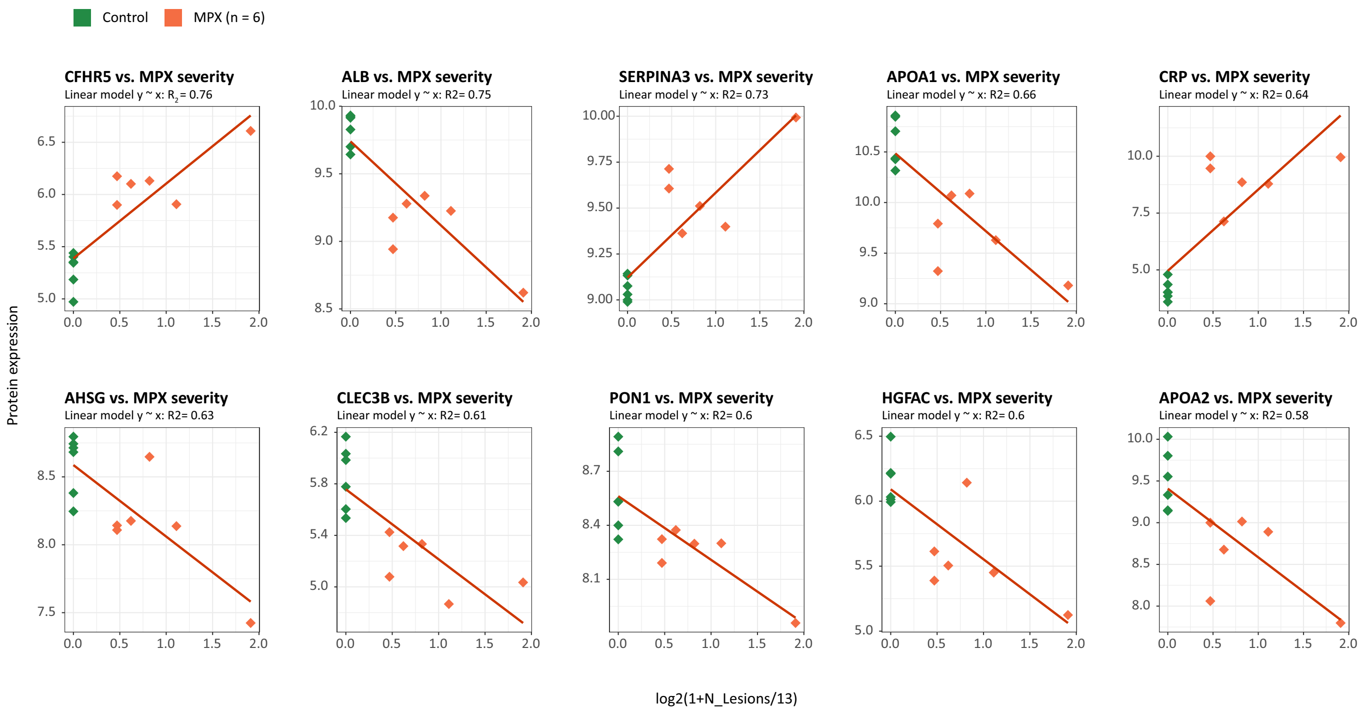

Figure EV3. Correlation between MPX (n = 6) severity (x-axis) and protein expression (y-axis).

As a measure of MPX severity the log2(1 + NLesions/13) was used. Here NLesions is the number of lesions. R^2 shows squared correlation coefficient. 6 MPX patients are colored orange, age matched Control patients are colored green. Fitted linear dependences of protein expression vs disease severity (here log2(1 + N_Lesion/Nmean), Nmean = 13) is shown as red line. Top 10 proteins with highest R^2 are shown.

© 2022 The Authors

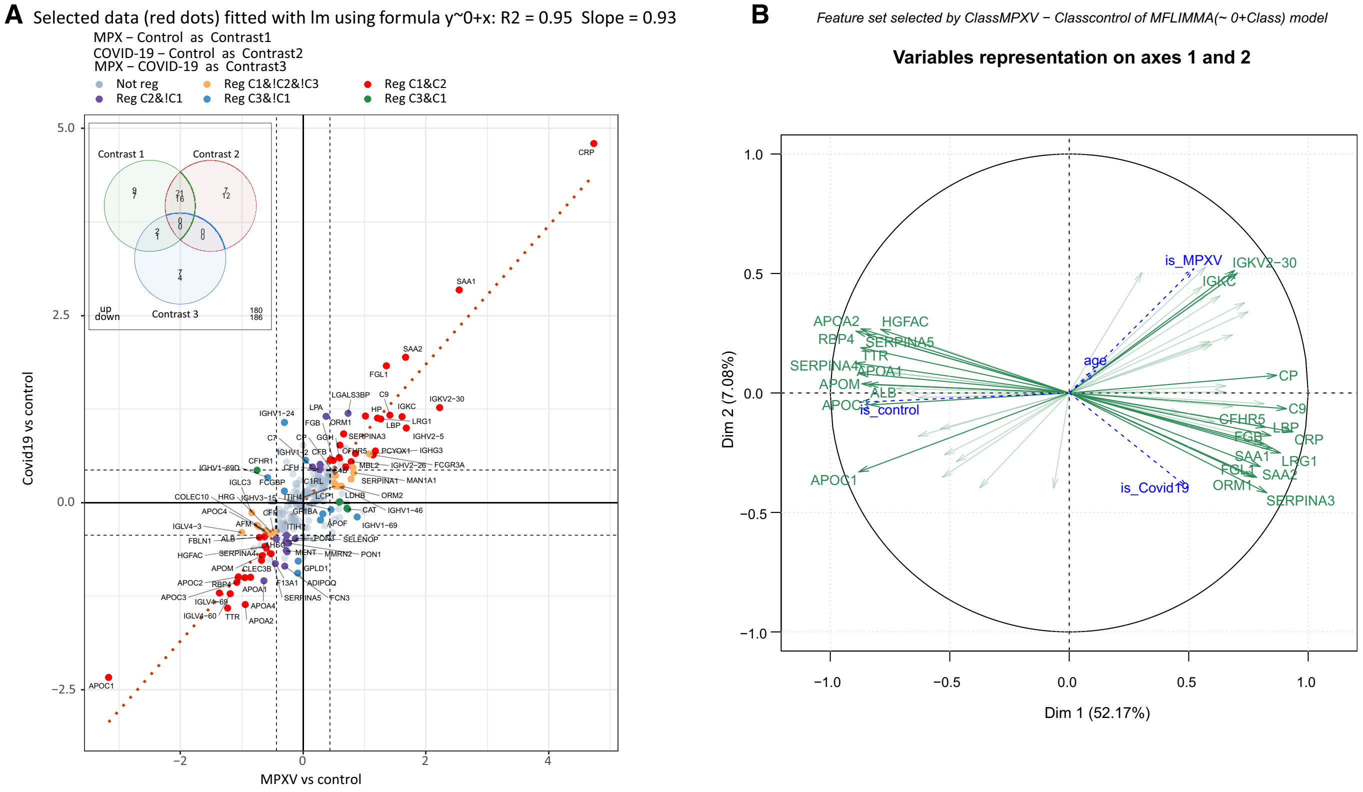

Figure EV4. Correlation of proteins regulation in contrasts MPX vs Control and COVID-19 vs Control.

A Scatterplot of logFC for contrast MPX vs Control (C1, x-axis) and logFC for contrast COVID-19 vs Control (C2, y- axis). Regulated proteins are color coded. Red color corresponds to (37) proteins regulated in both contrasts C1 and C2. There are no intersections between contrasts C2 and C3 (MPX vs. COVID-19). Orange group is for proteins specific for contrast C1 only (16 proteins). Green color is for proteins regulated both in contrast C1 and in contrast C3 (3 proteins). Blue color is for proteins regulated in C3, but not in C1 (11 proteins). And the pink color is for proteins regulated in C2 only (19 proteins). Red dotted line is linear regression through red dots, i.e. proteins regulated in C1 and C2. Note that orange and pink points have the same direction of regulation in both contrasts, C1 and C2. Only green and blue ones (except three proteins, ADIPOQ, GPLD1 and IGHV1-2) have opposite directions in C1 and in C2. Insert shows Venn diagram for intersection of up- and downregulated proteins between three contrasts.

B PCA loading plot based on proteins regulated in contrast MPX vs Control. It can be seen that despite the proteins were selected for MPX vs Control, the first principal component is in the direction of MPX + COVID-19 vs. Control and so the majority of regulated proteins are.

 © 2022 The Authors
