# Supplementary material for: The human host response to monkeypox infection: a proteomic case series study
Source: EMBO Mol Med. 2022 Sep 28;14(11):e16643. doi: 10.15252/emmm.202216643 (PMC9641420; doi:10.15252/emmm.202216643)
Supplement: Supplementary file 3 — Table EV2 [file EMMM-14-e16643-s001.docx]

|  | **MPX cases (n=6)** | | **healthy controls (n=15)** | | **COVID-19 controls (n=10)** | |
| --- | --- | --- | --- | --- | --- | --- |
| **male, n (%)** | 6 | 100 % | 15 | 100 % | 10 | 100 % |
| **age, years** | 31 | 27-41; 26-49 | 31 | 26-45; 23-50 | 39.5 | 25-47.5; 21-50 |
| **BMI, kg/m^2^** | 22.0 | 19.6-23.4; 17.6-25.1 | 23.1 | 21.2-24.9; 18.6-26.5 | 26.8 | 21.1-31.1; 20.0-39.2 |
| **TableEV 2**: Control cohort characteristics. COVID-19 controls were hospitalized without need of supplemental oxygen therapy.  Data are presented as median and IQR; range, unless otherwise specified. BMI: body mass index | | | | | | |
