# Supplementary material for: The human host response to monkeypox infection: a proteomic case series study
Source: EMBO Mol Med. 2022 Sep 28;14(11):e16643. doi: 10.15252/emmm.202216643 (PMC9641420; doi:10.15252/emmm.202216643)
Supplement: Supplementary file 5 — PDF+ [file EMMM-14-e16643-s003.pdf]

Report

TRANSPARENT PROCESS
OPEN ACCESS

EMBO Molecular Medicine

The human host response to monkeypox infection: a proteomic case series study

Ziyue Wang[1,†], Pinkus Tober-Lau[2,†], Vadim Farztdinov[3,†], Oliver Lemke[1], Torsten Schwecke[1], Sarah Steinbrecher[2], Julia Muenzner[1], Helene Kriedemann[2], Leif Erik Sander[2,4], Johannes Hartl[1], Michael Mülleder[3,†], Markus Ralser[1,4,5,*,†] & Florian Kurth[2,†]

Abstract

The rapid rise of monkeypox (MPX) cases outside previously endemic areas prompts for a better understanding of the disease. We studied the plasma proteome of a group of MPX patients with a similar infection history and clinical manifestation typical for the current outbreak. We report that MPX in this case series is associated with a strong plasma proteomic response among nutritional and acute phase response proteins. Moreover, we report a correlation between plasma proteins and disease severity. Contrasting the MPX host response with that of COVID-19, we find a range of similarities, but also important differences. For instance, CFHR1 is induced in COVID-19, but suppressed in MPX, reflecting the different roles of the complement system in the two infectious diseases. Of note, the spatial overlap in response proteins suggested that a COVID-19 biomarker panel assay could be repurposed for MPX. Applying a targeted protein panel assay provided encouraging results and distinguished MPX cases from healthy controls. Hence, our results provide a first proteomic characterization of the MPX human host response and encourage further research on protein-panel assays in emerging infectious diseases.

Keywords host response; monkeypox virus; proteomics; viral infection
Subject Categories Microbiology, Virology & Host Pathogen Interaction; Proteomics
DOI 10.15252/emmm.202216643 | Received 25 July 2022 | Revised 9 September 2022 | Accepted 9 September 2022 | Published online 28 September 2022
EMBO Mol Med (2022) 14: e16643

Introduction

The outbreak of monkeypox (MPX) with currently more than 40,000 confirmed infections worldwide, is exceptional in scale and spread (Kraemer *et al*, 2022), and has been declared a global emergency by the WHO (World Health Organisation, 2022a). MPX is caused by the zoonotic monkeypox virus (MPXV), a member of the genus *Orthopoxvirus* (World Health Organisation, 2022b). The first human MPX case was reported in 1970 in the Democratic Republic of the Congo (DRC), which is still the region with the highest level of endemicity in Africa (Bunge *et al*, 2022). Several outbreaks have been reported from African countries during the past decades, but research on MPX has largely been neglected. The clinical presentation often includes typical skin lesions, fever, and swollen lymph nodes. MPX is usually self-limiting, but severe cases can occur and a case fatality rate of 1–10% has been reported from Africa, with generally higher case fatality associated with infections from the Central African viral clade compared to the West African virus clade (Bunge *et al*, 2022).

The molecular epidemiology of the current MPX outbreak suggests that the current strain is closely related to that of a 2018–2019 outbreak in the United Kingdom and may have been circulating in the human population for some time, possibly with adaptation to the human host (Isidro *et al*, 2022; World Health Organisation, 2022c). In the current outbreak, there is a clear predominance of infections among men who have sex with men (MSM), and several large public events have been associated with the rapid emergence of cases in different parts of the world. Currently, transmission via close skin and mucosal contact, possibly including sexual transmission, seems likely (Dye & Kraemer, 2022; European Centre for Disease Prevention and Control, 2022; Pfäfflin *et al*, 2022; Thornhill *et al*, 2022). Even though the current outbreak is still in its early stages, a self-limiting course cannot be assumed; rather, it is a longer-term public-health problem that will hopefully bring diagnostic and therapeutic benefits to endemic African countries.

The COVID-19 pandemic has reminded us of the need to create infrastructure and methodologies to respond rapidly to emerging pathogens. Mass spectrometry-based proteomics is one of the emerging technologies in this regard, which due to the technical and analytical advances during the last years is increasingly moving into clinical applications (Liotta *et al*, 2001; Messner *et al*, 2020; Struwe *et al*, 2020; He *et al*, 2022). In the early phase of the COVID-19 pandemic, proteomic analyses provided rapid insights into the nature of

1 Department of Biochemistry, Charité – Universitätsmedizin Berlin, Berlin, Germany
2 Department of Infectious Diseases and Respiratory Medicine, Charité – Universitätsmedizin Berlin, Berlin, Germany
3 Core Facility High Throughput Mass Spectrometry, Charité – Universitätsmedizin Berlin, Berlin, Germany
4 Berlin Institute of Health, Berlin, Germany
5 The Wellcome Centre for Human Genetics, Nuffield Department of Medicine, University of Oxford, Oxford, UK
*Corresponding author. Tel: +49 30 45052814; E-mail: markus.ralser@charite.de
†These authors contributed equally to this work

© 2022 The Authors. Published under the terms of the CC BY 4.0 license.

the human response to SARS-CoV-2 and captured hallmarks of its immune evasion strategies and pathophysiology, including its impact on the complement system, coagulation cascade, and inflammatory and nutritional response machinery (D'Alessandro *et al*, 2020; Messner *et al*, 2020; Shen *et al*, 2020; Demichev *et al*, 2021; Overmyer *et al*, 2021; Nuñez *et al*, 2022). Furthermore, proteomic signatures turned out to classify disease severity in COVID-19 and allow for outcome prediction weeks in advance (Völlmy *et al*, 2021; Demichev *et al*, 2022; Nuñez *et al*, 2022). Recently, we were able to show the strength of mass spectrometry-based proteomics for rapid translation to medical care by generating a routine-applicable proteomic biomarker panel which predicted COVID-19 severity and outcome in a multicohort study (Wang *et al*, 2022a). While such proteomic assays are currently primarily used to monitor clinical trials, they are increasingly being considered for their potential to optimize treatment and resource allocation, as well as to aid navigation of difficult triaging situations in the event of a pandemic.

Here, we describe the proteomic changes in a case series, a small but characteristic group of patients hospitalized due to MPXV infection that share a similar disease and infection history. We detect significant and consistent proteomic changes caused by MPXV infection, enabling us to characterize the MPX host response at the proteomic level despite the moderate cohort size of a case series, in a timely manner. We report several protein markers that correlate with disease severity in the tested cases, that classify the disease proteome, and that contrast the human host response of MPXV to that of SARS-CoV-2 infection. Because we detected a partial overlap between the MPX and COVID-19 host response proteome, we also used a targeted proteomic panel developed for COVID-19 (Wang *et al*, 2022a) to explore the possibility of repurposing existing biomarker panels for classifying newly emerging infections. Although our results are derived from a small number of cases, they nonetheless suggest that repurposing of multiplex panel assays might be a viable strategy to improve pandemic preparedness. Our case series study provides a biochemical characterization of the MPX host response and reveals correlation of host proteins with MPX disease severity, and expands knowledge on protein panel testing for emerging infections.

Results

MPX patient case series and clinical presentation

A group of five patients were hospitalized at Charité University Hospital between 26th and 31st May 2022 for treatment of MPX, detected by PCR from cutaneous blisters. Interestingly, all patients had attended the same social event 10–14 days before developing symptoms, three of whom considered it most likely to have been infected on that occasion. We then included a 6th patient with an unrelated infection history who was hospitalized in mid-June 2022, but that otherwise had a related disease history. All six patients were of European descent, and all self-identified as men having sex with men (MSM) having practiced receptive anal sexual intercourse within 14 days prior to hospitalization. The group of patients was therefore notably homogeneous regarding history and time course of infection, triggering our interest in a case series study.

Overall, MPX patients exhibited mild to moderate symptoms, and no severe systemic affections such as encephalitis, myocarditis, or kidney failure were observed. Prodromes included fever, myalgia, and fatigue, and had already subsided in all patients by the time of admission to the hospital. The number of MPX skin lesions ranged from 5 to 36 and there were no clinical or laboratory signs of organ dysfunction. In all patients, the chief complaint and cause of hospitalization was severe anal or perianal pain requiring systemic analgesics in addition to topical treatment. Samples for proteome measurements were taken at a median of 8 days after symptom onset. Comorbidities included HIV ($n = 2$, both well controlled on antiretroviral therapy), other STIs ($n = 1$), and hepatitis C ($n = 1$). Patients were discharged with alleviated symptoms after 3–6 days. A summary of clinical characteristics is given in Table 1.

The partial sequence of the genome of the MPXV isolate obtained from one of the patients was determined and is available on GenBank (ON813251.2).

To gain maximum information from the case series cohort, we assembled two control cohorts. The first consisted of 15 age- and sex-matched healthy volunteers (Table EV2). Ten patients with SARS-CoV-2 infection, hospitalized due to moderate COVID-19 (grade 3 on the 8-point WHO ordinal scale, i.e., without the need for supplemental oxygen therapy), constituted the second control group. Their proteomes were measured within the same batch on our MS platforms, but had also been analyzed by us as part of a previous study (Demichev *et al*, 2021).

Table 1. Patient characteristics.

	MPX cases ($n = 6$)	
Male, *n* (%)	6	100%
Age, years	31	IQR: 27–41; range: 26–49
BMI, kg/m^2	22.0	IQR: 19.6–23.4; range: 17.6–25.1
Comorbidities, *n* (%)	3	50%
HIV, *n* (%)	2	33%
Hepatitis C, *n* (%)	1	17%
Other STIs[a], *n* (%)	1	17%
Δ symptom onset to sample, days	8	IQR: 5–14; range: 5–17
Δ PCR to sample, days	3.5	IQR 1.5–5; range: 0–5
Fever, *n* (%)	6	100%
Number of lesions	9	IQR: 5–20; range: 5–36
Duration of hospital stay, days	3.5	IQR: 3–5; range: 3–6
C-reactive protein at admission, mg/l	20.0	IQR: 10.4–57.9; range: 8.7–120.8
Leukocytes at admission, per nl	9.7	IQR: 8.3–11.7; range: 8.1–12.9
Lymphocytes at admission, per nl	3.1	IQR: 1.6–3.7; range: 1.4–3.8
Lactate dehydrogenase, U/l	214	IQR: 203–273; range: 181–381

BMI, body mass index; HIV, human immunodeficiency virus infection; STI, sexually transmitted infection.
Data are presented as median and IQR; range, unless otherwise specified.
[a]Other STIs: co-infection with Neisseria gonorrhoeae, Ureaplasma, and Mycoplasma hominis.

 © 2022 The Authors

A plasma proteomic signature of MPXV infection

Because of the moderate size of the case series study, we focused on obtaining maximally precise proteomic measurements and contrasted against both control groups. For obtaining proteomic measurements, we prepared tryptic digests from the MPX cases, matched healthy controls, and patients with moderate COVID-19, and included a broad panel of stable-isotope-labeled internal standards (PQ500, Biognosys). The tryptic digests obtained were then recorded using an online coupling of microflow chromatography and Zeno SWATH DIA, a latest generation of DIA proteomic technology (preprint: Wang *et al*, 2022b). Indeed, to our knowledge, the present study represents the first biomedical application of Zeno SWATH MS. After data were recorded as a single batch, raw data were processed with DIA-NN (Demichev *et al*, 2020), and data were post-processed to detect differentially concentrated proteins as well as the enrichment of pathway terms using pathway definitions from REACTOME (Croft *et al*, 2014). A workflow diagram of the procedures is provided (Fig 1A).

Considering the relatively mild severity of clinical symptoms and skin manifestation, the data revealed a substantial proteomic response to MPXV infection within the abundant "functional fraction" of the plasma proteome. This proteome fraction constitutes more than 99% of the plasma proteomic mass and is composed of around 300 proteins, most of which directly function in the plasma (Anderson & Anderson, 2002). As 200–300 of them are consistently quantified using high-throughput proteomics in neat plasma (Messner *et al*, 2020), and because this fraction contains more than 50 typical protein biomarkers (Demichev *et al*, 2021) that capture host physiological parameters (Vernardis *et al*, 2022), this functional fraction of the plasma proteome is of special interest for the development of clinical assays (Wang *et al*, 2022a). After pre-processing,

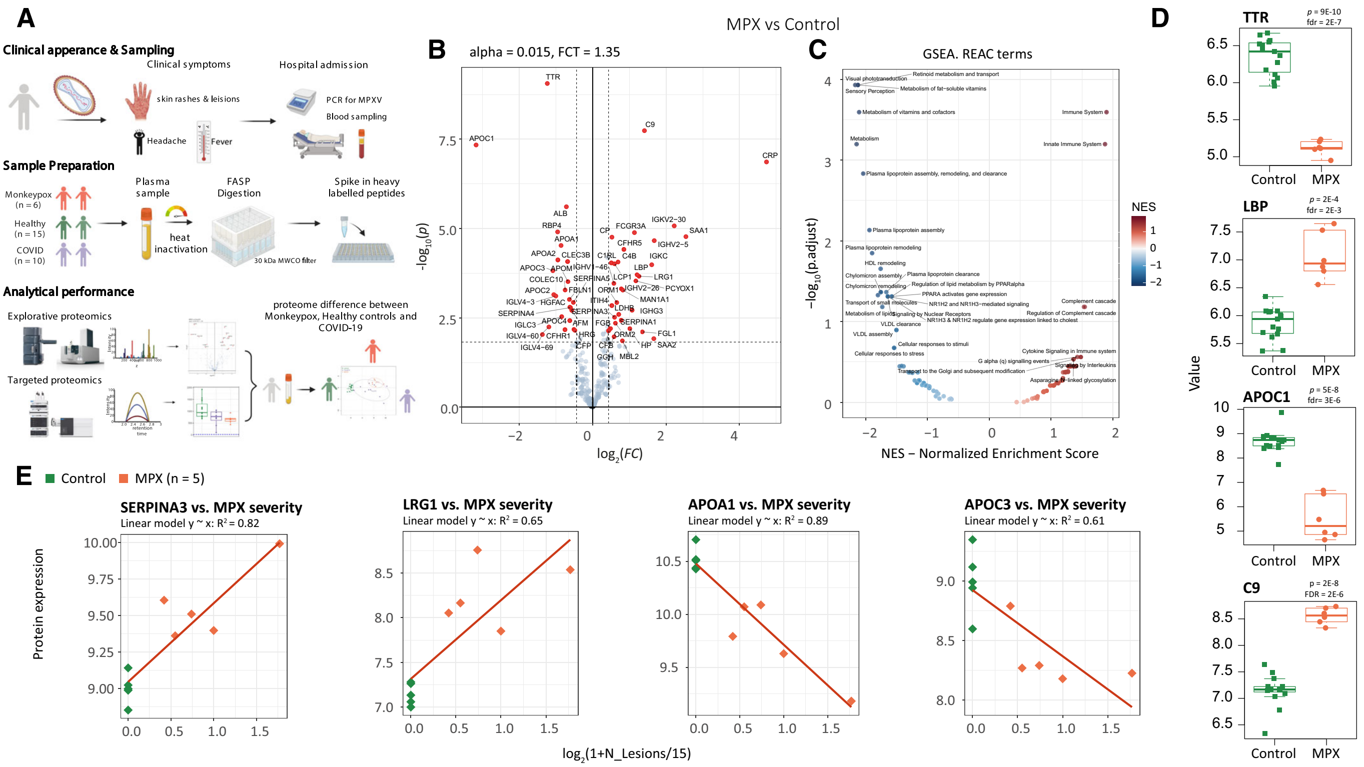

Figure 1. The human host response to monkeypox virus infection determined at the level of the plasma proteome.

A Schematic overview of the workflow using discovery proteomics (Zeno SWATH MS (Wang *et al*, 2022b)) in parallel to a targeted proteomic assay that quantifies COVID-19 severity biomarkers (Wang *et al*, 2022a) to characterize the plasma proteome in an MPX case series, and compare the proteomes to those of healthy volunteers and COVID-19 patients.

B Volcano plot of contrast MPX vs healthy controls; $\alpha <= 0.015$ and ¦logFC¦ >= 1.35 were used for selection of regulated proteins.

C Gene set analysis (GSEA) of REACTOME (Croft *et al*, 2014) terms enrichment for contrast MPX vs control. Y-axis shows $-\log_{10}$ of adjusted *P*-value (fdr) for Normalized Enrichment Score (x-axis) for each term. Terms with fdr <= 0.3 are labeled.

D Boxplots illustrating key proteins that differ between patients with MPX and controls (*P*-values and fdr for corresponding contrast MPX vs Control are provided in brackets): TTR (*P*-value = 9E-10, fdr = 2E-7), LBP (*P*-value = 2E-4, fdr = 2E-3), APOC1 (*P*-value = 5E-8, fdr = 3E-6), and C9 (*P*-value = 2E-8, fdr = 2E-6). Here, as usual, the central bar marks the median (second quartile), the bottom edge of the box marks the first quartile, the top edge of the box marks the third quartile, and the bottom and top whiskers mark the minimum and maximum values that are not outliers. The specific values of the protein expressions are also shown. Provided *P*-values are obtained from moderated statistics implemented in limma, dfrs were calculated according to Benjamini-Hochberg.

E Correlation between MPX severity ($N_{\text{Skin lesions}}$) and protein expression (y-axis). One MPX patient had an unclear additional skin condition (not a pure case of MPX) and therefore was excluded from the regression analysis that compares the number of skin lesions with the proteome; however, the proteome of this patient was largely in agreement with those of the other MPX cases (Fig EV3). As a measure of MPX severity, the $\log_2(1 + N_{\text{Lesions}} / 15)$ was used. Here N_{Lesions} is the number of lesions. R^2 shows squared correlation coefficient. MPX patients are colored orange, control patients green.

© 2022 The Authors

226 of the highly abundant proteins were found consistently quantified in the neat plasma sample. We detected low within-group coefficients of variation, below 25% for MPX and control, and about 34% for COVID-19 cases, indicating a high quantitative precision of the measurements, but also the presence of a biological signal (Fig EV1C). Indeed, we found 56 of the major plasma proteins to be differentially abundant in MPX patients compared to healthy controls. Twenty-four of these were lower concentrated in MPX, and 32 detected at a higher concentration (Fig 1B). The nature of the affected proteins indicated the molecular processes affected by MPX, as revealed by an enrichment analysis. For example, we see "immune system" and "regulation of complement cascade" mostly enriched among upregulated pathways. Among downregulated pathways, "plasma lipoprotein assembly" and "metabolism of fat-soluble vitamins" are enriched (Fig 1C).

At the level of individual proteins, the greatest differences between cases and controls were found in proteins associated with the acute phase response. These included significantly lower levels of the negative acute phase proteins TTR, ALB, and RBP4, as well as higher levels of acute phase proteins CRP, SAA1, SERPINA3, LBP, CP, and LRG1. Of note, various proteins involved in hepatic lipid metabolism and nutrient transport (APOA1, APOA2, APOC1, APOC2, APOC3) were lower in MPX patients than in controls, a known but not fully understood phenomenon also observed in other infections (Hardardóttir *et al*, 1995) (Fig 1D). Compared to controls, MPX patients exhibited a significantly higher level of complement component 9, the main element of the channel part of the membrane attack complex. Also, TTR in combination with the differentially expressed apolipoproteins is noteworthy, as it is a marker for malnutrition (Dellière *et al*, 2018), and we recently found it as a rapid responder in a caloric-restriction experiment conducted with healthy volunteers (Vernardis *et al*, 2022). We first speculated that acute MPX could result in a reduced caloric intake in affected patients. However, this picture was not confirmed by the clinical records of our patients, indicating that TTR is also part of the host response. We did not observe a significant influence of the concomitant conditions such as HIV or hepatitis C on the plasma proteomes. Results of the plasma proteomic response in patients with and without concomitant HIV infection are shown in Fig EV2. Both patients with HIV had immunologically well-controlled infections with suppressed viral load. Nevertheless, these patients can exhibit signs of ongoing immune activation, but if this response to HIV infection was present, it was masked by the acute response of the plasma proteome to the acute MPXV infection.

Next, we tested whether there is a relationship between the proteomic response and the number of skin lesions observed in our patients, determined as a proxy of disease severity. Several peptides showed a statistically robust correlation with the number of lesions, including the upregulated acute phase proteins SERPINA3, SAA1, and LRG1, as well as the downregulated apolipoproteins APOA1, APOA2, and APOC3 (Fig 1E). In particular, LRG1, an upstream modifier of TGF-beta signaling, is being increasingly recognized as an important contributor to disease pathogenesis and hence as a potential therapeutic target in a range of inflammatory conditions (Camilli *et al*, 2022). Despite the moderate size of the case series, our data suggests a consistent proteomic response in MPX cases that reflects the extent of skin manifestation and disease severity in MPX. Our case series did not contain severe cases. We can hence not predict the proteomic profile expected in severe cases, but our results suggest that with a more severe disease, stronger proteomic changes might become prevalent.

Relationship and intersection of the acute phase proteomic responses of MPX and COVID-19

The plasma proteome has similarly been shown to distinguish between different degrees of disease severity in other viral infections, including Ebola (Viodé *et al*, 2022) and COVID-19 (D'Alessandro *et al*, 2020; Shen *et al*, 2020; Demichev *et al*, 2021, 2022; Nuñez *et al*, 2022). To investigate to which degree this classification is due to a similar or divergent set of protein markers, we compared the MPX proteome response to that of an age- and sex-matched group of patients with moderately symptomatic COVID-19 (hospitalized, but without need of supplemental oxygen). The proteome obtained for these two patient groups revealed both an overlap in some response proteins and differences between the host responses against the two viral pathogens in other proteins. A simple hierarchical clustering based on Ward's agglomeration of Euclidean distances clearly separated healthy controls from MPX and COVID-19 cases (Fig 2A), and a protein expression analysis revealed differentially expressed proteins that are common between both diseases, but also those that differentiate the two infections from each other (Fig 2B (central part of the cloud), full-scale figure in Fig EV4A). Consistently, a principal component analysis (PCA) separated both patient groups (and controls), indicating that despite an overlap in several factors, the proteomes are discriminatory between MPX and COVID-19 (Fig 2C).

Contrasting the signatures at the protein level revealed that of the 56 proteins differentially expressed in MPX cases compared to healthy controls, 37 are also differentially expressed in COVID-19 patients with the same direction of regulation (Fig EV4A, Venn diagram). These include 12 proteins of the acute phase response such as SAA1 and LBP, and 12 proteins involved in coagulation, including FGB and SERPINA4, all of which have been found to be differentially expressed depending on COVID-19 disease severity.

Furthermore, we found 19 proteins that were differentially abundant in MPX but not in COVID-19. For instance, LCP1 (logFC(MPX-Control) = 0.6 ± 0.1, logFC(COVID-19-Control) = 0 ± 0.1), and LDHB (logFC(MPX-Control) = 0.7 ± 0.2, logFC(COVID-19-Control) = -0.1 ± 0.2) were found to be only upregulated in MPX (Fig 2D and E). LCP1 is interesting, because as L-plastin, it has been associated with membrane dynamics and the cytoskeleton and is an early tumor marker in kidney cancer (Ralser *et al*, 2005; Su Kim *et al*, 2013). Another protein that triggered our attention was CFHR1 (logFC(MPX-Control) = -0.8 ± 0.3, logFC(COVID-19-Control) = 0.4 ± 0.2), an inhibitor of the terminal pathway of the complement cascade, which was downregulated in MPX but was upregulated in COVID-19, where it is a marker of disease severity (D'Alessandro *et al*, 2020; Shen *et al*, 2020; Demichev *et al*, 2021). Indeed, hyperactivation of the complement system has been shown as a key feature for the pathophysiology of COVID-19 (Georg *et al*, 2022), but according to our proteome data, it is less important in MPX. Of note, it is plausible that additional differentially abundant proteins can be identified by proteomics in other or larger cohorts.

We deemed our case series too small to construct a robust classifier that identifies MPX cases on the basis of their proteome.

© 2022 The Authors

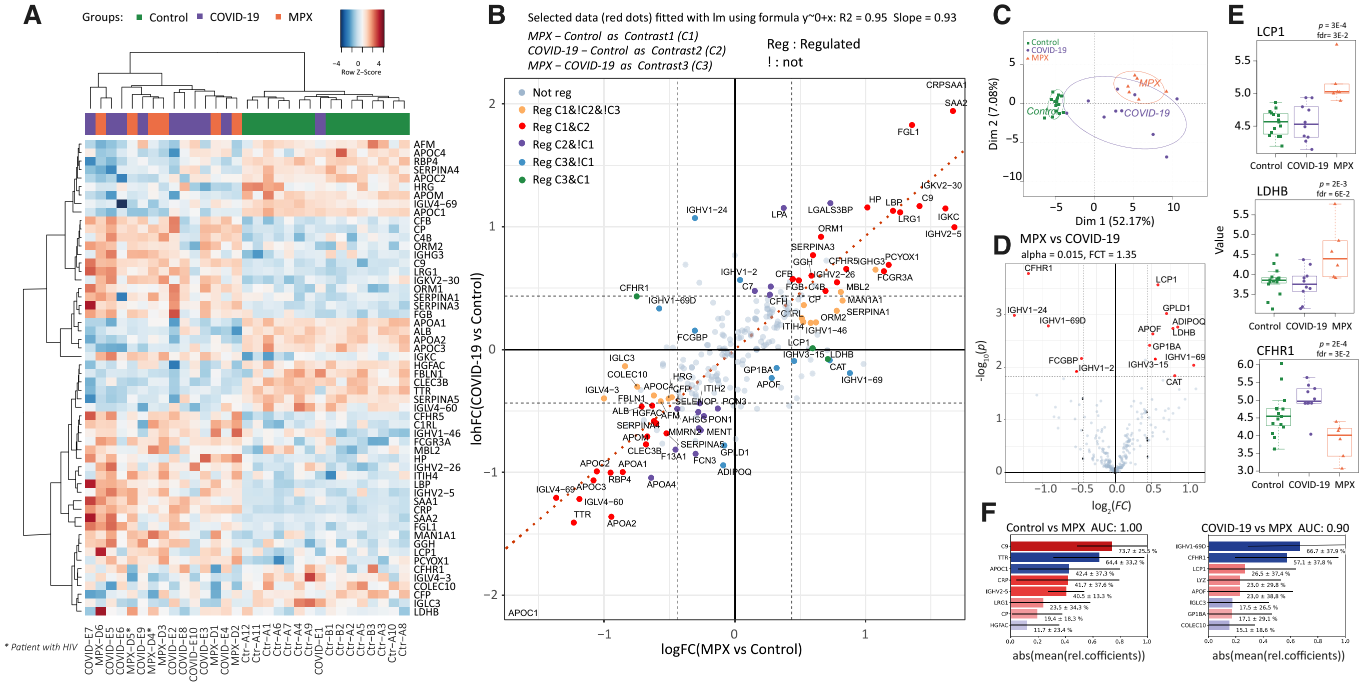

Figure 2. Differences and similarities between the plasma proteome upon infection with MPXV and SARS-CoV-2.

A Heatmap displaying hierarchical clustering using differentially regulated proteins between patients with MPX, COVID-19, and controls.

B Scatterplot of log fold-change (logFC) for contrast MPX vs control (C1, x-axis) and logFC for contrast COVID-19 vs control (C2, y-axis). Only the central part of the cloud is shown here. Three truncated dots (APOC1, CRP, and SAA1) are shown in the lower left and upper right corner. A full-scale figure is presented in Fig EV4A. Differentially abundant (Regulated; "Reg") proteins are color coded, with the red color corresponding to 37 proteins differentially abundant in both MPX vs control (C1) and COVID-19 vs control (C2), the orange color corresponding to proteins specifically changed in MPX vs control (C1) only (16 proteins), and the green color corresponding to proteins responding to both MPX vs control (C1) and MPX vs COVID-19 (C3) (3 proteins). There are no intersections between COVID-19 vs control (C2) and MPX vs COVID-19 (C3). The blue color corresponds to proteins responding to MPX vs COVID-19 (C3), but not in MPX vs control (C1) (11 proteins), and the pink color to proteins responding to COVID-19 vs control (C2) only (19 proteins). The red dotted line shows a linear regression through the red dots, i.e., proteins differentially abundant in MPX vs control (C1) and COVID-19 vs control (C2). Note that orange and pink points have the same direction of regulation in both MPXV vs control (C1) and COVID-19 vs control (C2). Only green and blue dots (except three proteins: ADIPOQ, GPLD1, and IGHV1-2) have opposite directions in C1 and in C2.

C *Post hoc* PCA score plot using proteins shown in (a).

D Differentially regulated proteins of patients with MPX and COVID-19 (Volcano Plot); α <= 0.015 and ¦logFC¦ >= 1.35 were used for selection of regulated proteins. The chosen significance level ensured that fdr for this contrast was below 22%.

E Key proteins that differ between patients with MPX and COVID-19 (Boxplots) (*P*-values and fdr for corresponding contrast MPX vs COVID-19 are provided in brackets): LCP1 (*P*-value = 3E-4, fdr = 3E-2), LDHB (*P*-value = 2E-3, fdr = 6E-2), CFHR1 (*P*-value = 2E-4, fdr = 3E-2). Here the central bar marks the median (second quartile), the bottom edge of the box marks the first quartile, the top edge of the box marks the third quartile, and the bottom and top whiskers mark the minimum and maximum values that are not outliers. The specific values of the protein expressions are also shown. Provided *P*-values are obtained from moderated statistics implemented in limma, dfrs were calculated according to Benjamini-Hochberg.

F Top 8 proteins of an SVM-trained model discriminating between healthy controls (n = 15) and MPX cases (n = 6) (left) or COVID-19 (n = 10) and MPX (n = 6) cases (right). Means of the relative coefficients over a 5-fold cross-validation are shown. Error bars denote the standard deviations. Red denotes positive, blue denotes negative coefficients. The AUC was calculated based on withheld samples that were not used for training the model.

However, we explored our data to see whether such an approach should be encouraged, and used a machine learning classifier to complement PCA and differential protein expression analysis, in the characterization of the MPX host response. Therefore, we tested a strictly cross-validated classifier to distinguish between MPX cases and healthy controls, as well as between MPX and COVID-19 cases on the basis of their proteomes. Within our data set, we achieved for both cases a differentiation with a high AUC on the test data that were withheld during training (Fig 2F). Encouragingly, the top-ranked differentiators identified by the machine-learning algorithm were also among the most differentially expressed proteins, like C9 (logFC(MPX-Control) = 1.4 ± 0.2) and TTR (logFC(MPX-Control) = −1.2 ± 0.1) for differentiating MPX cases and healthy individuals, or CHFR1 (logFC(MPX-COVID-19) = −1.2 ± 0.3) or LCP1 (logFC (MPX-COVID-19) = 0.6 ± 0.1) for differentiating MPX from COVID-19, respectively (Fig 2F). We note that due to the limited number of MPX cases in the case series, we can currently not validate the transferability of the model to other data and cohorts. Our data suggests however that the construction of such models appears feasible, once larger cohort data is available. It is further noteworthy that longitudinal proteome analysis in COVID-19 revealed a spike in proteomic response in the early disease phase, triggered by the inflammatory response, and that this early response signature was most predictive of outcome (Demichev *et al*, 2021). These results suggest that future studies should also follow the proteomic response to MPX in a longitudinal fashion.

© 2022 The Authors

Hence, our data provide a differentiated picture of the acute proteomic response that follows the two viral infections. On the one hand, we describe various acute phase proteins responding to both COVID-19 and MPX; on the other hand, both viral infections exhibit distinct proteomic response patterns, for instance, concerning the activation of the complement system. Hence, proteomics was effective in obtaining valuable insights even from a case series study.

Potential to repurpose proteomic assays to rapidly respond to emerging viral infections

Due to the partial overlap between the COVID-19 and MPX host responses, we speculated that there might be a potential to repurpose COVID-19 biomarker panel tests to MPX. We recently demonstrated the translational potential of plasma proteomics for applicability in clinical practice through the transfer of protein marker candidates which had been identified by discovery proteomics in COVID-19 into a routinely applicable targeted protein panel assay. The assay absolutely quantifies up to 50 peptides derived from 30 COVID-19-related plasma biomarker proteins and captured hallmarks of COVID-19 in a multi-cohort observational study conducted using routine-lab-compatible high-flow chromatography and LC-MRM acquisition (Wang *et al*, 2022a). The LC–MRM assay consistently quantified 32 of the peptides in plasma samples from MPX cases, controls, and in COVID-19 patient samples. Despite the assay being developed to quantify COVID-19 severity, a PCA on the peptides quantified also separated MPX patient samples from controls (Fig 3A). Moreover, a hierarchical clustering of the protein quantities that differed between healthy controls and MPX cases classified the disease samples (Fig 3B). This separation was driven by differential plasma levels of several proteins involved in the inflammatory and immune-mediated host response, e.g., increased levels of SERPINA3 and LYZ, or decreased levels of TF, TTR, HRG, PGLYRP2, and APOA1 (Fig 3C). Based on this proteomics data, we tested a classifier that distinguished between MPX cases and healthy controls within our data set to obtain a feature importance (Fig 3D). The most important features of the classifier (e.g., SERPINA3, AFM, PGLYRP2, and TF) overlapped with the differentially concentrated proteins in the COVID-19 plasma samples. Hence, within the limitations that our study is based on a small case series, our data suggest there is sufficient overlap among host response proteins among different viral diseases so that upon the disease specific adaptation of the statistical models, the biomarker panel assay could be adapted and repurposed to different viral infections, for instance, to improve pandemic preparedness.

Discussion

As case numbers rise, the current knowledge gap on the molecular etiology of MPX—a disease that has been known in central Africa

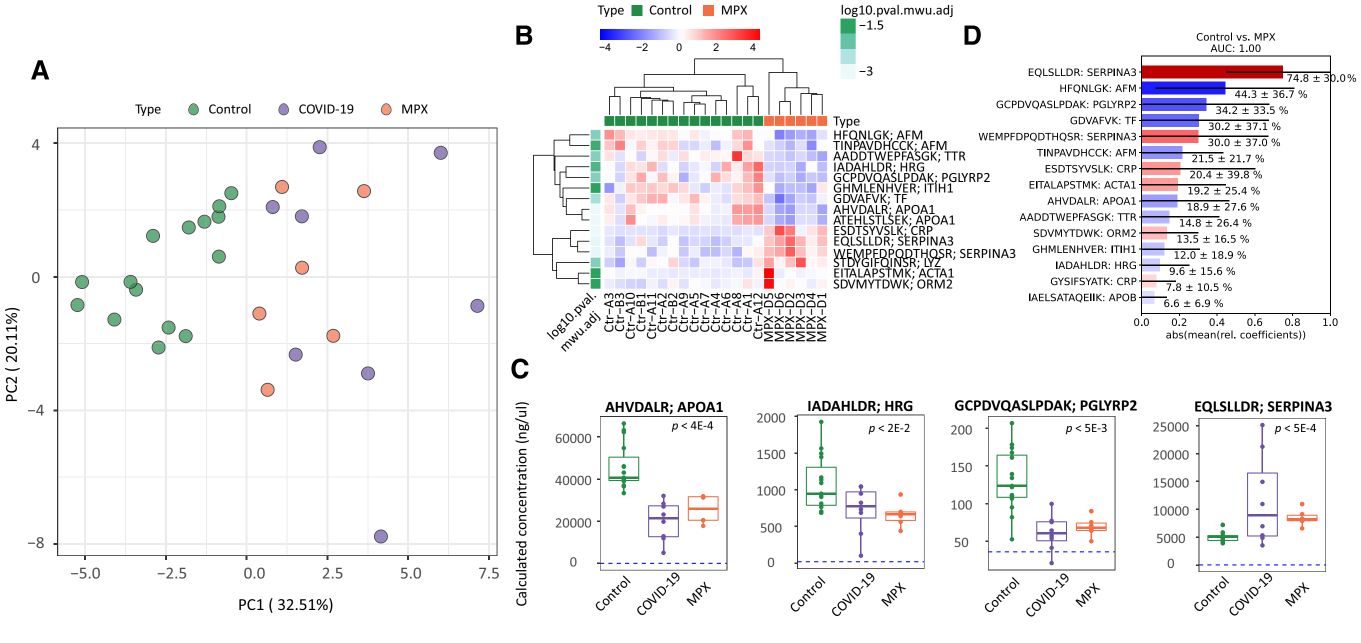

Figure 3. A targeted, multi-protein panel assay developed for COVID-19 infection discriminates patients with MPX from controls.

A Principal component analysis (PCA) of controls, patients with MPX, and COVID-19 with 32 peptides absolutely quantified in all samples using liquid chromatography selective reaction monitoring (LC-SRM).

B Hierarchical clustering using differentially regulated proteins between patients with MPX and controls (Heatmap); $P < 0.05$ with Mann–Whitney U test with FDR-based multiple testing correction.

C Key proteins that differ between patients with MPX and controls, and COVID-19 (Boxplots). Dashed blue lines indicate the lowest detected peptide concentration from calibration curves. Statistics based on Mann–Whitney U test with multiple testing corrections. For boxplots, median is indicated by a solid line, hinges show the 25th and 75th percentiles, whiskers show values that, at maximum, are within 1.5 times the interquartile range.

D Top 15 peptides of an SVM-trained model discriminating between healthy controls (n = 15) and MPX cases (n = 6). Means of the relative coefficients over a 5-fold cross-validation are shown. Error bars denote the standard deviations. Red denotes positive, blue denotes negative coefficients. The AUC was calculated based on withheld samples that were not used for training the model.

 © 2022 The Authors

for more than 50 years—becomes ever more apparent and calls urgently for a better understanding of this disease. In this context, a case series of individuals with similar demographics, timing, and course of disease who likely contracted the infection at the same social event caught our attention. Usually, the host response to a viral pathogen would be investigated in larger cohorts. However, considering urgently needed data and the parallel disease history of our case series, we speculated that because of the homogeneous and representative nature of cohort considering the current outbreak, even a low number of individuals may provide a clear proteomic signal, allowing us to provide a timely assessment of the host response to MPXV infection.

Indeed, analyzing the host response of the MPX patients at the proteome level provided a surprisingly clear picture, even in this small cohort, especially when comparing the proteomes to age- and sex-matched healthy individuals or patients with severity-matched, moderate COVID-19. Our dataset showed increased levels of specific acute phase proteins and overall lower nutritional response proteins such as TTR and apolipoproteins in MPX when compared to healthy controls. However, key pathways altered in COVID-19, including the complement and coagulation systems, were affected to a much lesser extent. The proteomic response described in our study therefore reflects the different pathophysiology connected with MPXV and SARS-CoV-2 as well as the mild to moderate disease severity in MPX observed in the current outbreak so far (Pfäfflin *et al*, 2022; Thornhill *et al*, 2022). Additional cohort studies will be required to validate our results in the broader context. Reassuringly however, most proteins identified by our non-targeted proteomic technique to be differentially abundant in MPX, have a known biological role in the acute phase response to viral infections. The correlation of numerous of the inflammatory proteins with disease severity gives additional and orthogonal confidence in our results.

We identified several peptides that showed a statistically robust correlation with disease severity as determined by the number of skin lesions. Organ dysfunction and severe disease have so far only sporadically been reported in the current outbreak in Europe and the US (Thornhill *et al*, 2022). MPX is however known to cause severe and lethal disease in endemic regions in Africa, with reported case fatality of up to 10% (Bunge *et al*, 2022).

Emerging pathogens with pandemic potential require fast responses, and an attractive possibility to achieve that is in the repurposing of existing procedures, diagnostics, and therapies, whenever possible. Prognostic biomarker panel assays were discussed during the COVID-19 pandemic for the monitoring of clinical trials, for supporting clinical decisions, and for their potential to support the navigation through difficult triaging situations (Struwe *et al*, 2020; Papadopoulou *et al*, 2022; Wang *et al*, 2022a). Drawing from our previous experience and based on the signature of the MPX human host response in discovery proteomics in the present study, we explored the application of a biomarker panel designed to classify patients with COVID-19 in routine laboratories (Wang *et al*, 2022a) on this very different viral disease. Within the limitations of a small case series study, the biomarker panel captured hallmarks of MPXV infection and facilitated a classification of patients with MPX and healthy controls in our sample set using an SVM model. The attractiveness of MRM panel assays is that they can be implemented in clinical workflows and are of low cost per sample. A panel of severity markers could be of help in endemic regions and possibly help to elucidate the pathophysiological differences between the Central African and West African clade of MPXV in the future. Our case series was too small to determine if the biomarker panel can be used to predict disease features, e.g., time to recovery, or to discriminate the effectiveness of therapeutic options. However, the correlation of the proteomic response with the number of skin lesions suggests that a predictive application of proteomics is possible for MPX and suggests conducting respective cohort studies in the near future. Indeed, we hope that the clear proteomic signature revealed by our case series justifies larger studies involving different cohorts and longitudinal sample in in the near future.

We believe our study demonstrates two essential aspects which are important for pandemic preparedness. First, our study exemplifies that when time is of the essence, proteomics can deliver valuable information on the molecular disease etiology of a moderate number of affected individuals, at least when their disease history is homogeneous and/or representative as in our case series study. Our results therefore imply that plasma proteomics might be particularly valuable for rare and neglected diseases, where proteomics may become an increasingly attractive toolkit for systemic analyses, despite limited case numbers. Indeed, given that symptoms were relatively mild, the proteomic host response to MPXV was distinct, with about one quarter of the highly abundant functional fraction of the plasma proteome changing. Second, our data suggested that there could be an untapped potential in the repurposing of biomarker panel assays across viral disease: although the overall proteomic signature clearly distinguished MPX from COVID-19, there was a sufficient overlap in the host response signature, so that we could distinguish MPX patients from healthy controls on the basis of a COVID-19 proteomic panel assay. Indeed, although our data hence shows that the individual biomarkers are not specific to a particular infection, the pattern in which they respond seems highly discriminatory. Although these results are to be regarded preliminary due to the moderate size of our cohort, our data suggests that one could generate a proteomic panel assay that is applicable across different viral diseases; in case of a new viral agent, one could hence measure the same panel of biomarkers, and only would need to adapt the data analysis and ML models to the novel agent. Future studies are needed to substantiate the viability of this possibility.

Materials and Methods

Patient cohort, biosamples, and clinical data

Patients with PCR-confirmed MPXV infection were recruited in a prospective observational study on the clinical and molecular characteristics of MPX. Written informed consent for collection of clinical data and blood was obtained from all patients before inclusion. Biosampling for proteomic measurements was performed on day 1–3 after admission to the hospital. Clinical data were captured in a purpose-built database. The study was approved by the ethics committee of Charité—Universitätsmedizin Berlin (EA2/139/22) and conducted in accordance with the Declaration of Helsinki and guidelines of Good Clinical Practice (EMA, 1996/2018). Biosamples for the cohort of patients with COVID-19 were obtained from the PaCOVID-19 study, a prospective observational cohort study on the

© 2022 The Authors

pathophysiology of COVID-19 conducted at Charité—Universitätsmedizin Berlin (Kurth *et al*, 2020; Thibeault *et al*, 2021). Biosamples for the cohort of healthy controls were obtained from a clinical study including healthy volunteers (Hillus *et al*, 2021).

Reagents and consumables

Water was from Merck (LiChrosolv LC–MS grade; Cat# 115333), acetonitrile was from Biosolve (LC–MS grade; Cat# 012078), trypsin (sequence grade; Cat# V511X) and trypsin/LysC mix (mass-spec grade; Cat# V5072) were from Promega, 1,4-dithiothreitol (DTT; Cat# 6908.2) was from Carl Roth, urea (puriss. P.a., reag. Ph. Eur.; Cat# 33247), Tris(2-carboxyethyl) phosphine hydrochloride (TCEP; Cat# 646547), and RIPA buffer (Cat# R0278) were from Merck, ammonium bicarbonate (ABC; eluent additive for LC–MS; Cat# 40867), 2-chloroacetamide (Cat# 22788), and dimethyl sulfoxide (DMSO; Cat# 41648) were from Fluka, formic acid (LC–MS grade; eluent additive for LC–MS; Cat# 85178), PCR sealing foil sheets (Cat# AB-0626), and Pierce quantitative fluorometric peptide assays (Cat# 23290) were from Thermo Fisher Scientific, bovine serum albumin (BSA; albumin Bovine Fraction V, Very Low Endotoxin, Fatty Acid-free; Cat# 47299) was from Serva, 96-well ultrafiltration plates (AcroPrep) Advance Filter Plates for Ultrafiltration, 1 ml, Omega 30 K MWCO (Cat# 8165) were from PALL, 96-well LoBind plates (Cat# ER0030129512-25EA) were from Merck, stable isotopic labeled (SIL) reference peptides for discovery proteomics (PQ500 Reference Peptides) were from Biognosys.

Sample preparation

Plasma samples were diluted 1:10 in RIPA buffer and heated at 95°C for 10 min. After cooling to room temperature (RT), 15 µl (~ 100 µg protein) were processed by FASP as previously described with minor modifications (Fossati *et al*, 2021) and transferred to a 96-well ultrafiltration plate mounted onto a collection plate (96-well LoBind plate). Liquid was removed by centrifugation (30 min, 1,800 × rcf, 20°C). Samples were denatured and reduced in 50 µl TUA buffer (8 M urea, 20 mM ammonium bicarbonate, 5 mM TCEP) for 30 min at room temperature without shaking. Following thiol alkylation (addition of 10 µl CA buffer (50 mM 2-chloroacetamide, 20 mM ABC) and incubation in the dark at RT for 30 min), the plate was centrifuged (30 min, 1,800 × rcf, 20°C). Samples were washed twice (30 min, 1,800 × rcf, 20°C) with 100 µl 20 mM ABC. Following an additional centrifugation to remove residual liquid (60 min, 1,800 × rcf, 20°C), the filter plate was moved to a fresh collection plate. To each well 50 µl 20 mM ABC containing 1 µg of trypsin/LysC mix was added, the plate was sealed with an adhesive PCR sealing foil sheet, and incubated at 37°C for 15 h. Peptides were collected by centrifugation (30 min, 1,800 × rcf, 20°C). Following the addition of 70 µl of HPLC-grade water to each well, the plate was centrifuged once more. The collection plate was then placed in a SpeedVac and samples were evaporated to complete dryness. Peptides were reconstituted in formic acid (30 µl, 0.1% v/v). Peptide concentration was determined using the Pierce quantitative fluorometric peptide assay.

For discovery proteomics, all samples (QCs, monkeypox, COVID-19, and healthy controls) were diluted to 200 ng/µl. The stable isotopic labeled reference peptides (PQ500 Reference Peptides) stock was prepared as described in the vendor's protocol (PQ500™ Reference Peptides Kit for Human Samples MANUAL), and diluted 1:10 in 50/50 v/v ACN:H2O. 2 µl of diluted PQ500 stock solution were spiked into 18 µl of the 200 ng/µl sample before transfer to vials for injection. For targeted proteomics, 15 µl of pre-digested heavy labeled standards (details in Wang *et al*, 2022a) were spiked into 10 µl samples (QCs, monkeypox, COVID-19, and healthy controls) and 20 µl were injected into the LC–MS system.

Mass spectrometry

Discovery proteomics using Zeno SWATH MS (preprint: Wang *et al*, 2022b) Tryptic digests were analyzed on a 7600 ZenoTOF mass spectrometer system (SCIEX), coupled to an ACQUITY UPLC M-Class system (Waters). 2 µl of each sample (360 ng sample + 0.02 µl PQ500, Biognosys) were loaded on a HSS T3 column (300 µm × 150 mm, 1.8 µm, Waters) heated to 35°C, then chromatographically separated with a 20-min gradient using a flow rate of 5 µl/min (Zelezniak *et al*, 2018). A Zeno SWATH acquisition scheme with 85 variable-size windows and 11-ms accumulation time with 1.4 s cycle time was used (preprint: Wang *et al*, 2022b) which allows for MS detection for average 7 points per chromatographic peak with the chosen chromatography.

Targeted proteomics by multiple reaction monitoring (plasma biomarker panel; Wang et al, 2022a)

Tryptic digests were analyzed on a 6495C triple quadrupole mass spectrometer (Agilent) coupled to a 1290 Infinity II UHPLC system (Agilent). Prior to MS analysis, samples were chromatographically separated on an InfinityLab Poroshell 120 EC-C18 column (2.1 × 50 mm, 1.9 µm, Agilent) heated to 45°C with a flow rate of 800 µl/min. The 6495C mass spectrometer was controlled by MassHunter Workstation software (LC–MS/MS Data Acquisition for 6,400 series Triple Quadrupole, Version 10.1 (Agilent)) and was operated in positive electrospray ionization mode. Samples were analyzed in dynamic multiple reaction monitoring (MRM) mode with both quadrupoles operating at unit resolution (Wang *et al*, 2022a).

Data processing

Discovery proteomics

The Zeno SWATH raw proteomics data was processed using DIA-NN (Demichev *et al*, 2020), 1.8.1 beta 20, availabe on github (DIA-NN github repository). The MS2 and MS1 mass accuracies were set to 20 and 12 ppm, and the scan window to 7. For the discovery approach, we used a publicly available spectral library for human plasma (Bruderer *et al*, 2019) and replaced spectra and RT information with DIA-NN in silico prediction. Protein inference was switched off and the match-between-runs (MBR) option was enabled. The processing pipeline is available in Supplementary Materials.

Targeted proteomics

LC–MRM data were processed using MassHunter Quantitative Analysis, v10.1 (Agilent). No blinding was done during peak integration. Peptide absolute concentration (expressed in ng/ml) was determined from calibration curves, constructed with native and SIL peptide standards in surrogate matrix (40 mg/ml BSA), and manually

© 2022 The Authors

validated. Linear regression analysis of each calibration curve was performed using custom R code (with 1/x weighting). Detailed information on transitions and matching of native peptides and internal standards can be found in Wang *et al* (2022a). Peptides with > 40% of values below the lowest or above the highest detected calibrant concentration across all samples were removed from analysis.

Data analysis

Clinical data analysis

Pseudonymized clinical data were processed using JMP Pro 16 (SAS Institute).

Discovery proteomics data analysis

Peptide expressions were first normalized within each clinical group. No blinding was done in normalization. To deal with a higher number of missing values in plasma proteomics compared to those obtained from cellular proteomics, we adopted the following approach: Peptides with excessive missing values (> 40% per group) were excluded from our analysis. This group-based thresholding delivered approximately the same number of peptides as the 26% presence threshold applied to the total set. The missing values of remaining peptides were imputed group-based using the PCA method (Josse & Husson, 2016). The group-based imputation allowed to avoid admixing of information from other groups. After imputation, an additional step of normalization was applied to the total set without using group information. In both cases, normalization was performed with LIMMA (Ritchie *et al*, 2015) implementation of cyclic loess method (Bolstad *et al*, 2003) with option "fast" (Ballman *et al*, 2004). To obtain a quantitative protein data matrix, the log2-intensities of peptides were filtered, only peptides belonging to one protein group were kept, and then summarized into protein log intensity using the PLM method (Bolstad, 2008, 41–59) implemented in the preprocessCore R package (Bolstad, 2021).

Statistical analysis of proteomics data was carried out in R using publicly available packages. Linear modeling was based on the R package LIMMA (Ritchie *et al*, 2015). The following model was applied to each tissue dataset (log2(p) is the log2-transformed expression of a protein): log2(p) ~ 0 + Class. The categorical factor Class had three levels: MPX, COVID-19, and control; reference level: control. For correlation between MPX severity (NSkin lesions) and protein expression, log2(1 + NLesions / 15) was used for linear regression. Log base 2 transformation was applied to bring the number of lesions to the same scale as protein expressions, 1 was added to guarantee that N lesions = 0 is transformed to 0, and division by Nmean = 15 (average number of lesions) was applied to map the average lesions number to 1. Also note that for 0 < N/Nmean < 1.5, deviations of f(N) from linearity are less than 12%.

For finding regulated features, the following criteria were applied for all contrasts: Significance level alpha was set to 0.015, which guaranteed the Benjamini–Hochberg (Benjamini & Hochberg, 1995) false discovery rate below 5% for contrast MPX vs Control, below 4% for contrast COVID-19 vs Control, and below 22% for contrast MPX vs COVID-19. The log fold-change threshold was applied to all contrasts to guarantee that the measured signal is above the average noise level. As such we took the median residual standard deviation of linear model: log2(T) = median residual SD of linear modeling

The paper explained

Problem

Until the recent outbreak, monkeypox was mainly confined to endemic areas in West and Central Africa, gaining little research interest. Aiming to breach the knowledge gap, we applied state-of-the-art plasma proteomics to a group of six patients with similar disease history and severity.

Results

Applying a recent proteomic method, ZenoSWATH-MS, on plasma samples obtained from a small but characteristic case series, we report distinct changes in proteins involved in the acute phase and nutritional response. Several proteins correlated with the number of skin lesions, indicating a potential use as disease severity markers. Comparing the proteomes to those of matched patients with COVID-19, we found numerous similarities. Moreover, we explored the usefulness of applying a proteomic COVID-19 biomarker panel assay to monkeypox cases and obtained a classification of the different disease groups.

Impact

This study is the first characterization of the human host response to monkeypox infection, offering insights into the pathophysiology. Moreover, we speculate that there is a thus far untapped potential for accelerating the response to disease outbreaks through the repurposing of biomarker assays.

(= log2(1.35)). Functional GSEA analysis was carried out using the clusterProfiler R package (Yu *et al*, 2012). For selecting the most (de)regulated pathway terms, we applied filter: 3 ≤ term size ≤ 300. The data matrix and description are provided in Dataset EV1.

Classifier construction and protein/peptide ranking

To complement the principal component and differential protein expression analysis, we constructed classifiers using a linear support vector machine (sklearn.svm.LinearSVC()) as implemented in scikit-learn 1.0.2 (Pedregosa *et al*, 2011) with an L1-penalty and balanced class-weights. The maximum number of iterations was increased to 10,000 to ensure convergence. As input, the log2-transformed quantities of the discovery proteomics and the 32 quantified peptides of the MRM panel were used, respectively.

The models were constructed and tested using a 5-fold shuffled and stratified cross-validation as implemented in sklearn.model_selection.StratifiedKFold(). For each iteration, 4 folds were used for training, 1 fold was used for testing the model. The data were scaled using sklearn.preprocessing.StandardScaler() fitted on the training data.

The AUC was calculated for the test data that were not used for training the model after all 5 iterations, resulting in one predicted value for every sample. For each iteration, the coefficients of the trained model were extracted and normalized by the maximum absolute coefficient of this iteration. For the plots, the mean and the standard deviation (error bars) of all 5 coefficients per protein/peptide were calculated and sorted according to the absolute mean. For reproducibility, the seed was fixed to 42.

Targeted proteomics

Significance testing of the absolute peptide concentrations and the sample type (control, MPX) was performed using Mann–Whitney *U*

© 2022 The Authors

test with multiple testing correction (where indicated). Test results are provided in Table EV1, *P*-values < 0.05 were considered significant.

Data availability

Internal patient IDs were changed at random within groups. Data matrix for discovery proteomics is available in (Dataset EV1). Proteomic raw data are deposited on PRIDE (https://www.ebi.ac.uk/pride/) under the project accession: PXD036074.

Expanded View for this article is available online.

Acknowledgments

Figure 1A and graphical abstract were made using BioRender (Biorender.com). We thank Vadim Demichev, Eva Tranter, Gisèle Godzick-Njomgang, Daniel Wendisch, Linda Jürgens, and Frieder Pfäfflin for their valuable input and help in collecting biosamples, and Hezi Tenenboim for proofreading our manuscript. We thank Christof von Kalle and the Charité-BIH Clinical Study Centre for their support in setting up our translational research platform. This research was funded in part by the Berlin Institute of Health (BIH), Wellcome Trust (IA 200829/Z/16/Z to MR), the European Research Council (ERC) under grant agreement ERC-SyG-2020 951475 (to MR), by the Ministry of Education and Research (BMBF), as part of the National Research Node Mass spectrometry in Systems Medicine (MSCoresys), under grant agreement 031L0220 (to MR), the Deutsche Forschungsgemeinschaft (DFG) (INST 335/797-1) (to MM) and the Berlin University Alliance (501_Massen-spektrometrie, 501_Linklab). We further acknowledge funding by Deutsche Forschungsgemeinschaft (DFG) supporting ZW's PhD studies as part of the SFB/TRR 186. Open access funding enabled and organized by Projekt DEAL.

Author contributions

Ziyue Wang: Formal analysis; visualization; methodology; writing—original draft; writing—review and editing. **Pinkus Tober-Lau:** Data curation; writing—original draft; writing—review and editing. **Vadim Farztdinov:** Formal analysis; visualization; writing—original draft; writing—review and editing. **Oliver Lemke:** Formal analysis; visualization; writing—original draft; writing—review and editing. **Torsten Schwecke:** Methodology; writing—original draft. **Sarah Steinbrecher:** Data curation. **Julia Muenzer:** Formal analysis; writing—original draft. **Helene Kriedemann:** Data curation. **Leif Erik Sander:** Project administration. **Johannes Hartl:** Data curation; formal analysis; methodology; writing—original draft; writing—review and editing. **Michael Mülleder:** Conceptualization; supervision; funding acquisition; methodology; writing—original draft; project administration; writing—review and editing. **Markus Ralser:** Conceptualization; supervision; funding acquisition; writing—original draft; project administration; writing—review and editing. **Florian Kurth:** Conceptualization; data curation; supervision; writing—original draft; project administration; writing—review and editing.

Disclosure and competing interests statement

The authors declare that they have no conflict of interest.

References

Anderson NL, Anderson NG (2002) The human plasma proteome: history, character, and diagnostic prospects. *Mol Cell Proteomics* 1: 845 – 867

Ballman KV, Grill DE, Oberg AL, Therneau TM (2004) Faster cyclic loess: normalizing RNA arrays via linear models. *Bioinformatics* 20: 2778 – 2786

Benjamini Y, Hochberg Y (1995) Controlling the false discovery rate: a practical and powerful approach to multiple testing. *J R I State Dent Soc* 57: 289 – 300

Bolstad B (2008) Preprocessing and Normalization for Affymetrix GeneChip Expression repoMicroarrays. In *Methods in Microarray Normalization*, P Stafford (ed), pp 41 – 59. Boca Raton, FL: Taylor & Francis

Bolstad B (2021) preprocessCore: a collection of pre-processing functions

Bolstad BM, Irizarry RA, Astrand M, Speed TP (2003) A comparison of normalization methods for high density oligonucleotide array data based on variance and bias. *Bioinformatics* 19: 185 – 193

Bruderer R, Muntel J, Müller S, Bernhardt OM, Gandhi T, Cominetti O, Macron C, Carayol J, Rinner O, Astrup A *et al* (2019) Analysis of 1508 plasma samples by capillary-flow data-independent acquisition profiles proteomics of weight loss and maintenance. *Mol Cell Proteomics* 18: 1242 – 1254

Bunge EM, Hoet B, Chen L, Lienert F, Weidenthaler H, Baer LR, Steffen R (2022) The changing epidemiology of human monkeypox-A potential threat? A systematic review. *PLoS Negl Trop Dis* 16: e0010141

Camilli C, Hoeh AE, De Rossi G, Moss SE, Greenwood J (2022) LRG1: an emerging player in disease pathogenesis. *J Biomed Sci* 29: 6

Croft D, Mundo AF, Haw R, Milacic M, Weiser J, Wu G, Caudy M, Garapati P, Gillespie M, Kamdar MR *et al* (2014) The Reactome pathway knowledgebase. *Nucleic Acids Res* 42: D472 – D477

D'Alessandro A, Thomas T, Dzieciatkowska M, Hill RC, Francis RO, Hudson KE, Zimring JC, Hod EA, Spitalnik SL, Hansen KC (2020) Serum proteomics in COVID-19 patients: altered coagulation and complement status as a function of IL-6 level. *J Proteome Res* 19: 4417 – 4427

Dellière S, Neveux N, De Bandt J-P, Cynober L (2018) Transthyretin for the routine assessment of malnutrition: A clinical dilemma highlighted by an international survey of experts in the field. *Clin Nutr* 37: 2226 – 2229

Demichev V, Messner CB, Vernardis SI, Lilley KS, Ralser M (2020) DIA-NN: neural networks and interference correction enable deep proteome coverage in high throughput. *Nat Methods* 17: 41 – 44

Demichev V, Tober-Lau P, Lemke O, Nazarenko T, Thibeault C, Whitwell H, Röhl A, Freiwald A, Szyrwiel L, Ludwig D *et al* (2021) A time-resolved proteomic and prognostic map of COVID-19. *Cell Syst* 12: 780 – 794

Demichev V, Tober-Lau P, Nazarenko T, Lemke O, Kaur Aulakh S, Whitwell HJ, Röhl A, Freiwald A, Mittermaier M, Szyrwiel L *et al* (2022) A proteomic survival predictor for COVID-19 patients in intensive care. *PLOS Digit Health* 1: e0000007

Dye C, Kraemer MUG (2022) Investigating the monkeypox outbreak. *BMJ* 377: o1314

EMA (1996/2018) ICH E6 (R2) Good clinical practice. In *European Medicines Agency* [Internet]. 17 Sep 2018. https://www.ema.europa.eu/en/ich-e6-r2-good-clinical-practice

European Centre for Disease Prevention and Control (2022) Joint ECDC-WHO Regional Office for Europe Monkeypox Surveillance Bulletin

Fossati A, Frommelt F, Uliana F, Martelli C, Vizovisek M, Gillet L, Collins B, Gstaiger M, Aebersold R (2021) System-wide profiling of protein complexes via size exclusion chromatography-mass spectrometry (SEC-MS). In *Methods in Molecular Biology*, JM Walker (ed), pp 269 – 294. Cham: Springer Nature

Georg P, Astaburuaga-García R, Bonaguro L, Brumhard S, Michalick L, Lippert LJ, Kostevc T, Gäbel C, Schneider M, Streitz M *et al* (2022) Complement activation induces excessive T cell cytotoxicity in severe COVID-19. *Cell* 185: 493 – 512

Hardardóttir I, Grunfeld C, Feingold KR (1995) Effects of endotoxin on lipid metabolism. *Biochem Soc Trans* 23: 1013 – 1018

© 2022 The Authors

He B, Huang Z, Huang C, Nice EC (2022) Clinical applications of plasma proteomics and peptidomics: towards precision medicine. *Proteomics Clin Appl* e2100097

Hillus D, Schwarz T, Tober-Lau P, Vanshylla K, Hastor H, Thibeault C, Jentzsch S, Helbig ET, Lippert LJ, Tscheak P *et al* (2021) Safety, reactogenicity, and immunogenicity of homologous and heterologous prime-boost immunisation with ChAdOx1 nCoV-19 and BNT162b2: a prospective cohort study. *Lancet* 9: 1255 – 1265

Isidro J, Borges V, Pinto M, Ferreira R, Sobral D, Nunes A, Dourado Santos J, José Borrego M, Núnci S, Pelerito A, *et al* (2022) First draft genome sequence of Monkeypox virus associated with the suspected multi-country outbreak, May 2022 (confirmed case in Portugal). *Virological*

Josse J, Husson F (2016) missMDA: a package for handling missing values in multivariate data analysis. *J Stat Softw* 70: 1 – 31

Kraemer MUG, Tegally H, Pigott DM, Dasgupta A, Sheldon J, Wilkinson E, Schultheiss M, Han A, Oglia M, Marks S *et al* (2022) Tracking the 2022 monkeypox outbreak with epidemiological data in real-time. *Lancet Infect Dis* 22: 941 – 942

Kurth F, Roennefarth M, Thibeault C, Corman VM, Müller-Redetzky H, Mittermaier M, Ruwwe-Glösenkamp C, Heim KM, Krannich A, Zvorc S *et al* (2020) Studying the pathophysiology of coronavirus disease 2019: a protocol for the Berlin prospective COVID-19 patient cohort (Pa-COVID-19). *Infection* 48: 619 – 626

Liotta LA, Kohn EC, Petricoin EF (2001) Clinical proteomics: personalized molecular medicine. *JAMA* 286: 2211 – 2214

Messner CB, Demichev V, Wendisch D, Michalick L, White M, Freiwald A, Textoris-Taube K, Vernardis SI, Egger A-S, Kreidl M *et al* (2020) Ultra-high-throughput clinical proteomics reveals classifiers of COVID-19 infection. *Cell Syst* 11: 11 – 24

Nuñez E, Orera I, Carmona-Rodríguez L, Paño JR, Vázquez J, Corrales FJ (2022) Mapping the Serum Proteome of COVID-19 Patients; Guidance for Severity Assessment. *Biomedicine* 10: 1690

Overmyer KA, Shishkova E, Miller IJ, Balnis J, Bernstein MN, Peters-Clarke TM, Meyer JG, Quan Q, Muehlbauer LK, Trujillo EA *et al* (2021) Large-scale multi-omic analysis of COVID-19 severity. *Cell Syst* 12: 23 – 40

Papadopoulou G, Manoloudi E, Repousi N, Skoura L, Hurst T, Karamitros T (2022) Molecular and clinical prognostic biomarkers of COVID-19 severity and persistence. *Pathogens* 11: 311

Pedregosa F, Varoquaux G, Gramfort A, Michel V, Thirion B, Grisel O, Blondel M, Prettenhofer P, Weiss R, Dubourg V *et al* (2011) Scikit-learn: Machine learning in Python. *J Mach Learn Res* 12: 2825 – 2830

Pfäfflin F, Wendisch D, Scherer R, Jürgens L, Godzick-Njomgang G, Tranter E, Tober-Lau P, Stegemann MS, Corman VM, Kurth F *et al* (2022) Monkeypox in-patients with severe anal pain. *Infection* https://doi.org/10.1007/s15010-022-01896-7

Ralser M, Nonhoff U, Albrecht M, Lengauer T, Wanker EE, Lehrach H, Krobitsch S (2005) Ataxin-2 and huntingtin interact with endophilin-A complexes to function in plastin-associated pathways. *Hum Mol Genet* 14: 2893 – 2909

Ritchie ME, Phipson B, Wu D, Hu Y, Law CW, Shi W, Smyth GK (2015) limma powers differential expression analyses for RNA-sequencing and microarray studies. *Nucleic Acids Res* 43: e47

Shen B, Yi X, Sun Y, Bi X, Du J, Zhang C, Quan S, Zhang F, Sun R, Qian L *et al* (2020) Proteomic and metabolomic characterization of COVID-19 patient sera. *Cell* 182: 59 – 72

Struwe W, Emmott E, Bailey M, Sharon M, Sinz A, Corrales FJ, Thalassinos K, Braybrook J, Mills C, Barran P (2020) The COVID-19 MS Coalition—accelerating diagnostics, prognostics, and treatment. *Lancet* 395: 1761 – 1762

Su Kim D, Choi YD, Moon M, Kang S, Lim J-B, Kim KM, Park KM, Cho NH (2013) Composite three-marker assay for early detection of kidney cancer. *Cancer Epidemiol Biomarkers Prev* 22: 390 – 398

Thibeault C, Mühlemann B, Helbig ET, Mittermaier M, Lingscheid T, Tober-Lau P, Meyer-Arndt LA, Meiners L, Stubbemann P, Haenel SS *et al* (2021) Clinical and virological characteristics of hospitalised COVID-19 patients in a German tertiary care centre during the first wave of the SARS-CoV-2 pandemic: a prospective observational study. *Infection* 49: 703 – 714

Thornhill JP, Barkati S, Walmsley S, Rockstroh J, Antinori A, Harrison LB, Palich R, Nori A, Reeves I, Habibi MS *et al* (2022) Monkeypox virus infection in humans across 16 countries – April-June 2022. *N Engl J Med* 387: 679 – 691

Vernardis S, Demichev V, Lemke O, Grüning N-M, Messner C, White M, Pietzner M, Peluso A, Collet T-H, Henning E *et al* (2022) Acute caloric restriction acts on the plasma proteome and reveals Apolipoprotein C1 as a signal of nutritional state and metabolic disease. *Res Square*

Viodé A, Smolen KK, Fatou B, Wurie Z, Van Zalm P, Konde MK, Keita BM, Ablam RA, Fish EN, Steen H (2022) Plasma proteomic analysis distinguishes severity outcomes of human Ebola virus disease. *MBio* e0056722

Völlmy F, van den Toorn H, Zenezini Chiozzi R, Zucchetti O, Papi A, Volta CA, Marracino L, Vieceli Dalla Sega F, Fortini F, Demichev V *et al* (2021) A serum proteome signature to predict mortality in severe COVID-19 patients. *Life Sci Alliance* 4: e202101099

Wang Z, Cryar A, Lemke O, Tober-Lau P, Ludwig D, Helbig ET, Hippenstiel S, Sander L-E, Blake D, Lane CS *et al* (2022a) A multiplex protein panel assay for severity prediction and outcome prognosis in patients with COVID-19: an observational multi-cohort study. *eClinicalMedicine* 49: 101495

Wang Z, Mülleder M, Batruch I, Chelur A, Textoris-Taube K, Schwecke T, Hartl J, Causon J, Castro-Perez J, Demichev V, *et al* (2022b) High-throughput proteomics of nanogram-scale samples with Zeno SWATH DIA. *medRxiv* https://doi.org/10.1101/2022.04.14.488299 [PREPRINT]

World Health Organisation (2022a) Second meeting of the International Health Regulations (IHR) Emergency Committee regarding the multi-country outbreak of monkeypox

World Health Organisation (2022b) WHO Fact Sheet on Monkeypox

World Health Organisation (2022c) WHO Director-General's opening remarks at the COVID-19 media briefing– 1 June 2022

Yu G, Wang L-G, Han Y, He Q-Y (2012) clusterProfiler: an R package for comparing biological themes among gene clusters. *OMICS* 16: 284 – 287

Zelezniak A, Vowinckel J, Capuano F, Messner CB, Demichev V, Polowsky N, Mülleder M, Kamrad S, Klaus B, Keller MA *et al* (2018) Machine learning predicts the yeast metabolome from the quantitative proteome of kinase knockouts. *Cell Syst* 7: 269 – 283

License: This is an open access article under the terms of the Creative Commons Attribution License, which permits use, distribution and reproduction in any medium, provided the original work is properly cited.

Expanded View Figures

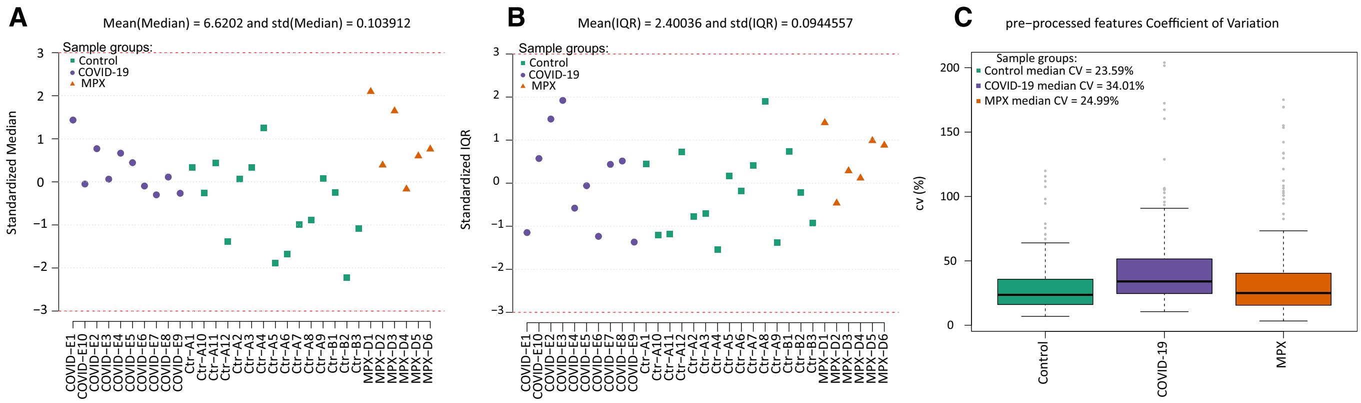

Figure EV1. Samples quality control (QC).

A QC chart of standardized median expression of proteins the study sample.
B QC chart of standardized interquartile range (IQR) of protein expressions in the study samples.
C Box plot of within group coefficient of variation of protein intensities.

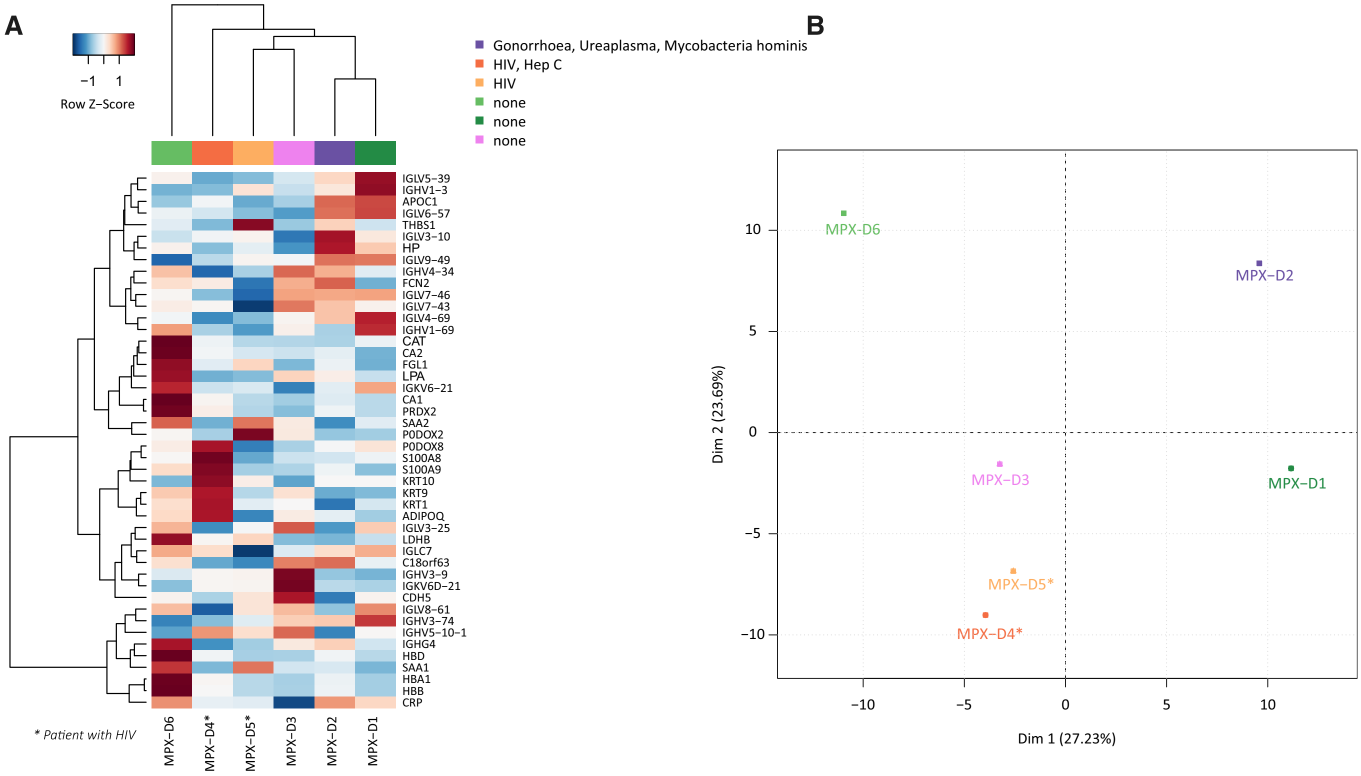

Figure EV2. Exploratory analysis of monkeypox samples clustering according to comorbidities.

A Hierarchical clustering using top 20% of most variable proteins. It is seen that HIV samples do not cluster together.
B PCA score plot using full proteome. It is seen that HIV is not a driving factor of samples variance. Its largest contribution is into the second principal component.

 © 2022 The Authors

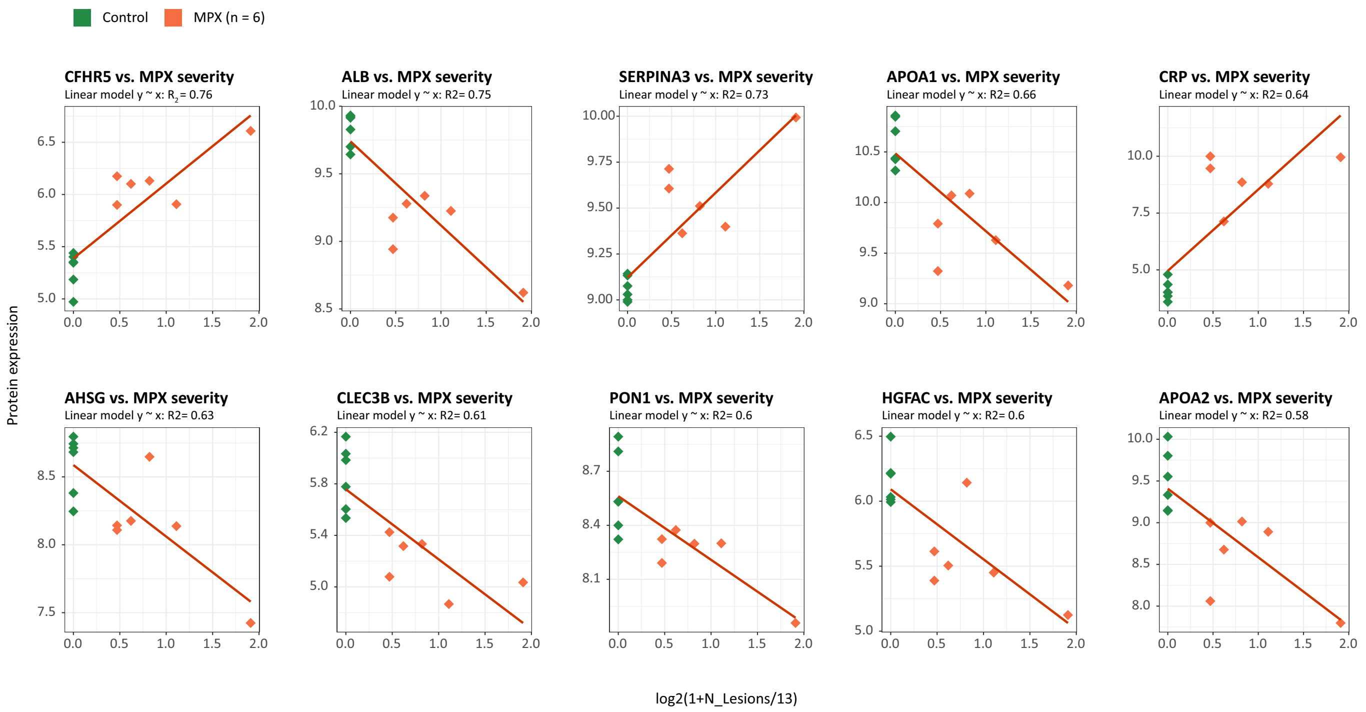

Figure EV3. Correlation between MPX (n = 6) severity (x-axis) and protein expression (y-axis).

As a measure of MPX severity the log2(1 + NLesions/13) was used. Here NLesions is the number of lesions. R^2 shows squared correlation coefficient. 6 MPX patients are colored orange, age matched Control patients are colored green. Fitted linear dependences of protein expression vs disease severity (here log2(1 + N_Lesion/Nmean), Nmean = 13) is shown as red line. Top 10 proteins with highest R^2 are shown.

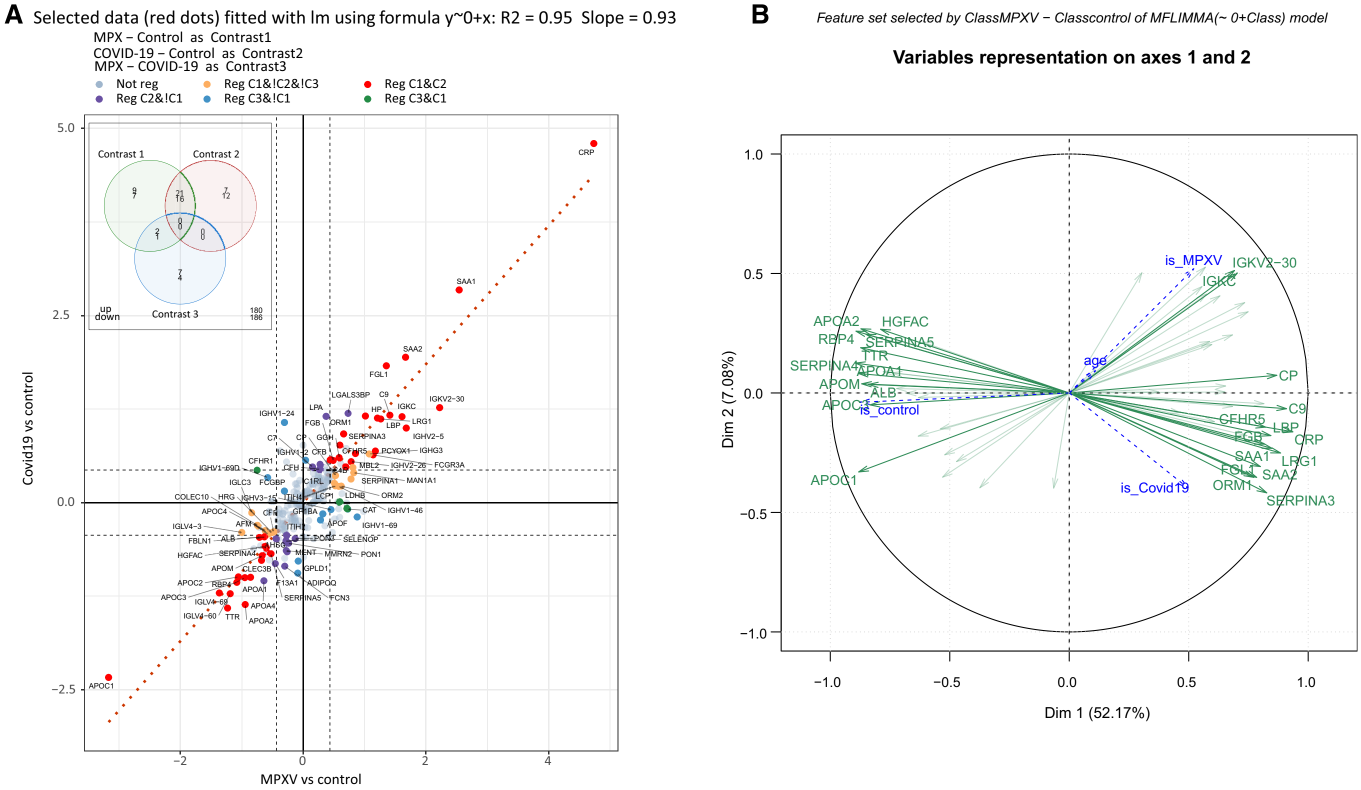

Figure EV4. Correlation of proteins regulation in contrasts MPX vs Control and COVID-19 vs Control.

A Scatterplot of logFC for contrast MPX vs Control (C1, x-axis) and logFC for contrast COVID-19 vs Control (C2, y- axis). Regulated proteins are color coded. Red color corresponds to (37) proteins regulated in both contrasts C1 and C2. There are no intersections between contrasts C2 and C3 (MPX vs. COVID-19). Orange group is for proteins specific for contrast C1 only (16 proteins). Green color is for proteins regulated both in contrast C1 and in contrast C3 (3 proteins). Blue color is for proteins regulated in C3, but not in C1 (11 proteins). And the pink color is for proteins regulated in C2 only (19 proteins). Red dotted line is linear regression through red dots, i.e. proteins regulated in C1 and C2. Note that orange and pink points have the same direction of regulation in both contrasts, C1 and C2. Only green and blue ones (except three proteins, ADIPOQ, GPLD1 and IGHV1-2) have opposite directions in C1 and in C2. Insert shows Venn diagram for intersection of up- and downregulated proteins between three contrasts.

B PCA loading plot based on proteins regulated in contrast MPX vs Control. It can be seen that despite the proteins were selected for MPX vs Control, the first principal component is in the direction of MPX + COVID-19 vs. Control and so the majority of regulated proteins are.

 © 2022 The Authors
