# Supplementary material for: The human host response to monkeypox infection: a proteomic case series study
Source: EMBO Mol Med. 2022 Sep 28;14(11):e16643. doi: 10.15252/emmm.202216643 (PMC9641420; doi:10.15252/emmm.202216643)
Supplement: Supplementary file 6 [file EMMM-14-e16643-s004.pdf]

EMBO Molecular Medicine

The human host response to monkeypox infection: a proteomic case series study

Ziyue Wang, Pinkus Tober-Lau, Vadim Farztdinov, Oliver Lemke, Torsten Schwecke, Sarah Steinbrecher, Julia Muenzer, Helene Kriedemann, Leif Sander, Johannes Hartl, Michael Mülleder, Markus Ralser, and Florian Kurth
DOI: 10.15252/emmm.202216643

Corresponding author: Markus Ralser (markus.ralser@charite.de)

Review Timeline:

Submission Date:	25th Jul 22
Editorial Decision:	9th Aug 22
Revision Received:	23rd Aug 22
Editorial Decision:	8th Sep 22
Revision Received:	9th Sep 22
Accepted:	9th Sep 22

Editor: Jingyi Hou

Transaction Report:

(Note: With the exception of the correction of typographical or spelling errors that could be a source of ambiguity, letters and reports are not edited. Depending on transfer agreements, referee reports obtained elsewhere may or may not be included in this compilation. Referee reports are anonymous unless the Referee chooses to sign their reports.)

9th Aug 2022

Thank you again for submitting your work to EMBO Molecular Medicine. We have now heard back from the three referees who agreed to evaluate your manuscript. As you will see from the reports below, the referees are overall supportive and think the study is interesting. Still, they raise a series of concerns, which we would ask you to address in a revision of the manuscript.

Without reiterating all the points raised in the reviews below, some of the more substantial issues are the following:

- Referees #2 and #3 raised concerns about the classifier analysis. Considering these comments, please either remove these results or add independent validation to better support the conclusion.
- Tone down the conclusion about repurposing COVID-19 biomarker panels and discuss the specificity concern of Referee #3.
- In light of Referee #2's suggestion, we would ask you to use "Reports" as manuscript type when you submit the revised version. More detailed guidelines regarding "Reports" can be found here https://www.embopress.org/page/journal/17574684/authorguide#reportsarticleguide.

All other issues raised by the referees need to be satisfactorily addressed. Please feel free to contact me in case you would like to discuss in further detail any of the issues raised by the referees.

We would welcome the submission of a revised version within three months for further consideration. Please note that EMBO Molecular Medicine strongly supports a single round of revision. As acceptance or rejection of the manuscript will depend on another round of review, your responses should be as complete as possible.

EMBO Molecular Medicine has a "scooping protection" policy, whereby similar findings that are published by others during review or revision are not a criterion for rejection. Should you decide to submit a revised version, I do ask that you get in touch after three months if you have not completed it to update us on the status.

We are aware that many laboratories cannot function at full efficiency during the current COVID-19/SARS-CoV-2 pandemic and have therefore extended our "scooping protection policy" to cover the period required for a full revision to address the experimental issues. Please let me know should you need additional time, and also if you see a paper with related content published elsewhere.

Please read below for important editorial formatting and consult our author's guidelines for proper formatting of your revised article for EMBO Molecular Medicine.

I look forward to receiving your revised manuscript.

Use this link to login to the manuscript system and submit your revision: https://embomolmed.msubmit.net/cgi-bin/main.plex

Kind regards,
Jingyi

Jingyi Hou
Editor
EMBO Molecular Medicine

*****

***** Reviewer's comments *****

Referee #1 (Comments on Novelty/Model System for Author):

na

Referee #1 (Remarks for Author):

A very interesting manuscript on a prevalent topic MPXV infection in which the authors report on the potential use and application of proteomic screening assays derived during the Covid pandemic on the MPXV endemic.
A few points to consider for the discussion section:

- With the very low number of MPXV patients included and the comparatively high number of chronic viral coinfections (HIV, HCV) additional wording is warranted to the results into place particularly in the light of still ongoing immune activation even in well controlled HIV infection (2/5 -> 40% of patients ...)
- Pls comment on why analyses at different timepoints (i.e. longitudinal) of the variable clinical course of MPXV infections were not done
- Authors should stress that their findings were derived from pts with mild to moderate disease (hospitalisation ocurred only for analgesia). What could be expected in pts with e.g. meningitis or other serious systemic affections?

Referee #2 (Comments on Novelty/Model System for Author):

The research question is interesting, but the sample size is relatively small (only 6 MPX patients). However, this study reports latest data for an ongoing MPX outbreak.

Referee #2 (Remarks for Author):

This study reports plasma proteomic data for six MPX patients and compared them to that from 15 healthy donors and 10 moderate COVID-19 patients. They used a cutting edge DIA method with ZenoTOF, and quantified ~300 proteins. Data analysis reveled the association between a few key proteins and the MPX phenotype. In general, this study is of high interest to the ongoing MPX outbreak, and offered unique insights to the disease pathogenesis. However, the main limitation is that the entire

study is based on data from six MPX patients, and the proteomic depth is not very high therefore many dysregualted proteins are not included in the analyses. Probably a short report in EMBO Mol Med might be more appropriate.

The manuscript is well written, but the authors may further improve it by considering the following major issues.

1) The machine learning part may not make lots of sense because it is based on 6 MPX patients, and there is no independent validation of the classifier. How could the authors exclude the possibility of overfitting? Either independent validation is required, or the authors may considering removing the statement of the classifier.

2) In the last section of Results, the authors claimed the potential to repurpose proteomic assays but this is based on only six patients, and largely hypothetical instead of presenting convincing data to support the claim. The reviewer suggests the authors to substantially tone down this claim and move some of the statement into Discussion.

Minor issues:
1) page 2: line 12: COVID19 --> COVID-19
2) page 7, around line 15: please clarify the threshold of adjusted p values and fold-change for determining significantly regulated proteins for all the analyses of identification of differentially expressed proteins.
3) Fig 1c: please explain why the p.adjust was set as 0.3
4) Fig 1d, Fig2e, Fig 3c: please add p values for these box plots.
5) fig2d: are p valued adjusted?

Referee #3 (Comments on Novelty/Model System for Author):

The model system is comprised of 5 patients diagnosed with Monkey Pox. Two of these patients also have HIV infections (though managed with minimal viral load). The authors have quantified 200-300 proteins in serum from these individuals and contrasted them against CoVID19 and age/gender matched non-infected/healthy controls. While they do observe significantly different features between the three datasets, the sample size and limited demographic range limit the power of this dataset to make strong conclusions. They also do some limited predictive modelling to support their observations which off a dataset of n=5, is not possible to judge the validity and should be removed.

Referee #3 (Remarks for Author):

This manuscript is from a highly proficient and established group performing blood-proteomic analysis on clinical samples. The work here shows changes to proteins that arise during infection from Monkey Pox Virus in contrast to COVID 19 and healthy controls. The laboratory has a strong-established platform and track record for this type of analysis. Investigations into Monkey Pox infections are incredibly relevant given the current ongoing pandemic across Africa. As acknowledged by the authors, the principal limitation is the sample size (n=5) which is extremely small and does not represent a wide demographic, especially in relation to African populations. The authors perform some predictive modelling, though the purpose of this is not clear - with n=5 it is very limited to what conclusions can be taken from these, and it may be sensible to remove them altogether. That said, the authors also make valid discussion on the re-purposing of a biomarker test, using one developed by the same group for stratifying COVID19 patients and demonstrated that it can also detect MPX infection. However, this raises further questions/concerns about the specificity of the tests as the key predictive proteins were the same. Given the current MPX epidemic, this analysis is important, however for publication in a molecular-medical journal, a greater number and relevant more relevant demographic (the disease is prevalent in Africa, this cohort are (presumably) European as they were hospitalised in Germany?) need to be included. Aside from these major considerations, several minor points are raised below that should be addressed. Reviewed by Harry Whitwell, Imperial College London.

MINOR
1. The first sentence is missing comma's:
"The outbreak of Monkey pox (MPX), with more than 15,000 confirmed infections worldwide, is exception...."
2. I think the last sentence in the first paragraph should be:
"with generally higher case fatality associated with infections from the Central African viral clade compared to the West African virus clade [4]."
3. The second sentence in 3rd paragraph - "application" should be "applications".
4. In methods - you allow peptides with up to 40% missing values; this seems like a lot and suggests a possible problem with the MS acquisition. Can you justify this high level of inclusion and suggest what caused the missing values? This should extend to a justification of the choice of imputation method.
In methods - can you justify the rational for the protein expression transformation: log2(1+Nlesions-/15)?
5. In methods (first paragraph in Discovery proteomics data analysis), I cannot find a reference to PLM in reference 35. And the Bioconductor page for the package does not contain a description either. Please give the full name and a suitable reference to describe what this is doing to derive protein-level expression data from the peptide values.
6. Table 1 - two ranges are given in the third column - what are these? Matched data for controls should be provided in the same table in the main text.

7. How were the MPX patients treated, was a sample taken before any hospital intervention?
8. Typo P11, line 23 "thia" should be "this".
9. How many quantitative points over each peptide LC peak? - this should be reported as you are aiming to "[obtain] maximally precise proteomics measurements". The CV values presented in the results (25% and 34%) - are these median CV across all peptides? How many peptides achieved this CV? The yaxis label for Sup Fig 1C is missing. How successful was the normalisations? The iRT was spiked into each sample, so could be compared across samples (should have minimal CV calculated within each batch and across all batches). Otherwise, it is not clear what the purpose of adding these was.
10. Fig2B axis should say log-FC to help with interpretation. Assuming that "!" mean "not" in the top legend - this nomenclature should be explained in the legend. In fact, the legends here are confusing and it took a while for me to get it. The two legends need to be next to each other to help the reader make sense of it. I would advise re-thinking the legend altogether to describe what each point means. I think there is probably space in the top left and bottom right corner of the plot to give a description of each point like how it is given in the legend. Especially as the figure is large (1page) so the legend may not be on the same page as the figure.
11. In results: "Furthermore, we found 19 proteins that were differentially abundant in MPX but not in COVID-19. For instance, LCP1 and LDHB were found to be only upregulated in MPX (Fig 2 d, e)" - fold change and p-value should be given to help with interpretation. The sample size is only small, therefore the power to detect small changes is probably very limited.
12. Results - the classifier between for MPX vs Control and MPX vs CoVID contains 8 proteins (fig 2f) (though 5 proteins are mentioned in the methods?) and is built based on 5 samples..... I'm not sure this adds anything - the sample size is limited (as acknowledged), in my view, this analysis does nothing to support the other observations. I would drop it.
13. I found the re-purposing of biomarker panels provocative and certainly worthy of inclusion for support of this important concept. The generation of a predictive model based on only 5 cases again is likely overfitted - but the point is to show that this panel may be repurposed rather than to propose a test directly. Therefore, I think as presented it is of interest. Of concern though is the specificity to differentiate between the diseases. In general, the fold-change difference between differentiating proteins is not great and could be due to chance. This needs further discussion to be convincing. SERPINA3 and APOA1 were both similarly changing in the discovery proteomics dataset for both MPX and COVID19 vs controls (red dots in Fig2b). Therefore, would this classifier be expected to differentiate between diseases? - probably not (you also show this in the classifier figures).
14. I think the last sentence in the results is missing something - I'm not following its meaning (perhaps should be "...in the `COVID19` disease plasma"?).

Point-by-point reply to the reviewers

Referee #1 (Remarks for Author):

A very interesting manuscript on a prevalent topic MPXV infection in which the authors report on the potential use and application of proteomic screening assays derived during the Covid pandemic on the MPXV endemic.

A few points to consider for the discussion section:

- With the very low number of MPXV patients included and the comparatively high number of chronic viral coinfections (HIV, HCV) additional wording is warranted to the results into place particularly in the light of still ongoing immune activation even in well controlled HIV infection (2/5 -> 40% of patients ...)

We thank the reviewer for the comment. Indeed, it is noteworthy that none of the patients with chronic viral co-infections showed different signatures than the other MPX cases. In addition to showing the specific individual proteomic profiles of the two patients living with HIV versus those of the other patients in FigureEV 2, we have now highlighted this in the results, as suggested by the reviewer.

"*We did not observe a significant influence of the concomitant conditions such as HIV or Hepatitis C on the plasma proteomes. Results of the plasma proteomic response in patients with and without concomitant HIV infection are shown in (FigureEV 2). Both patients with HIV had immunologically well-controlled infections with suppressed viral load. Nevertheless, these patients can exhibit signs of ongoing immune activation, but if this response to HI-virus infection was present, it was masked by the acute response of the plasma proteome to the acute MPXV infection.*"

- Pls comment on why analyses at different timepoints (i.e. longitudinal) of the variable clinical course of MPXV infections were not done

Due to the explorative character of this case study we decided to focus on samples taken early after admission to the ward, and we had no complete longitudinal sample follow-ups available. Nonetheless, we were able to identify distinct proteomic signatures from these single time point measurements. In a next step, we aim to further substantiate our findings in a larger cohort of patients including longitudinal characterization of the plasma proteome in monkeypox. We have added a sentence to the discussion, stating that future studies should address the proteomic response to monkeypox infection in a longitudinal fashion.

"*It is further noteworthy that longitudinal proteome analysis in COVID-19 revealed a spike in proteomic response in the early disease phase, triggered by the inflammatory response, and that this early response signature was most predictive of outcome (Demichev et al, 2021). These results suggest that future studies should also follow the proteomic response to MPX in a longitudinal fashion.*"

- Authors should stress that their findings were derived from pts with mild to moderate disease (hospitalisation occurred only for analgesia). What could be expected in pts with e.g. meningitis or other serious systemic affections?

Fortunately, so far the current outbreak of monkeypox is characterized by mild to moderate disease with severe disease courses being very rare and to date less than 10 deaths among over 40.000 cases. This is reflected in our case series, where we did not have patients thus far with serious systemic affections. Intriguingly, we identified proteomic markers that showed a correlation with the number of skin lesions, one clinical surrogate for disease severity. This suggests that severe cases may have a distinct proteomic response. We have added a short reference to the valuable point of the reviewer, that can unfortunately only be speculatively answered with the moderately affected patient cohort available to us.

"Overall, MPX patients exhibited mild to moderate symptoms, and no severe systemic affections such as encephalitis, myocarditis or kidney failure were observed."

"Moreover, it is noteworthy that consistent with the current outbreak, our case series did not contain severe cases. We can hence not comment on the proteomic profile expected in severe cases, but our results suggest that with a more severe disease, stronger proteomic changes might become prevalent."

Referee #2 (Comments on Novelty/Model System for Author):

The research question is interesting, but the sample size is relatively small (only 6 MPX patients). However, this study reports latest data for an ongoing MPX outbreak.

We completely agree with the Reviewer. We decided on publishing this case series study because it provides important information about an emerging viral outbreak in a timely manner. We highlight now more prominently in the discussion that our study doesn't render comprehensive studies with larger patient numbers obsolete, in fact, we hope it triggers larger studies: Indeed, part of the value of our study is precisely that it helps to initiate and justify larger studies - because it shows that a significant and scientifically valuable proteomic response is expected in MPX.

"Indeed, we hope that the clear proteomic signature revealed by our case series justifies larger studies involving different cohorts and longitudinal sampling in the near future."

Referee #2 (Remarks for Author):

This study reports plasma proteomic data for six MPX patients and compared them to that from 15 healthy donors and 10 moderate COVID-19 patients. They used a cutting edge DIA method with ZenoTOF, and quantified ~300 proteins. Data analysis reveled the association between a few key proteins and the MPX phenotype. In general, this study is of high interest to the ongoing MPX outbreak, and offered unique insights to the disease pathogenesis. However, the main limitation is that the entire study is based on data from six MPX patients, and the proteomic depth is not very high therefore many dysregualted proteins are not included in the analyses. Probably a short report in EMBO Mol Med might be more appropriate.

We thank the reviewer for the positive comments; we agree with the Reviewer and Editor that our study is a fit for the “report” format, and have changed it accordingly. The proteomic depth achieved corresponds to more than 99.9% of the plasma proteomic mass. It is correct that the plasma contains many additional, low abundant proteins, many of which leak from tissues, or are cell-cell signaling molecules. The attractiveness of measuring the high abundant, or functional, plasma proteome is however that it characterizes many processes that are key for the antiviral immune response, including the complement system, the coagulation system, or the Apolipoprotein class, and we deemed the chosen, state of the art proteomic method, hence ideal for an initial characterisation of the MPX host response at the proteome level.

The manuscript is well written, but the authors may further improve it by considering the following major issues.

1) The machine learning part may not make lots of sense because it is based on 6 MPX patients, and there is no independent validation of the classifier. How could the authors exclude the possibility of overfitting? Either independent validation is required, or the authors may considering removing the statement of the classifier.

We apologize if our writing caused a misunderstanding about the rationale for constructing a machine learning classifier in this particular case, and thank the reviewer for bringing this up: Likely other readers could have been confused as well and we hope to have improved the manuscript in this regard in the revision:

The reviewer is correct that it is unlikely that a machine learning classifier constructed on the basis of our small case series, might (universally or robustly) classify MPX patients (i.e. in other cohorts). In our case this is however not due to statistical issues (i.e. overfitting, see Methods), but because the biological variability is unlikely to be fully captured with just six cases. We completely agree with the Reviewer on this point.

However, following the argument ‘all models are wrong but some are useful’, there are two points that are valuable about our ML model: First, it provides a statistically independent, and perhaps more robust measure (next to the PCA and the simple differential expression (DE) analysis) showing that the proteomes of MPX patients are different to those of healthy controls. This is because we can cross-validate the ML model, and despite the moderate case number we could split the data in a training and prediction set. Second, with the ML model we can rank the discriminatory features, and hence get othogonal information about which proteins classify the MPX infection.

To address the reviewer’s fully justified point, we have toned down on the application ML model and have re-written the text to the best of our knowledge, so that the rationale for constructing a ML classifier for testing the separation of the proteomes becomes more clear.

“*We deemed our case series too small to construct a robust classifier that identifies MPX cases on the basis of their proteome. However, we explored our data to see whether such an approach should be encouraged in larger cohorts, and moreover, used a machine learning classifier to complement PCA and differential protein expression analysis, in the characterisation of the MPX host response.* ”

2) In the last section of Results, the authors claimed the potential to repurpose proteomic assays but this is based on only six patients, and largely hypothetical instead of presenting convincing data to support the claim. The reviewer suggests the authors to substantially tone down this claim and move some of the statement into Discussion.

We agree with the reviewer's comment that this, although reasonable, is a speculative point. We have rephrased the results section to specify that we applied a COVID-19 biomarker assay to this cohort, yielding results which in our view merit further investigation and potentially indicate the possibility to repurpose these assays in the future.

"*Based on this proteomics data, we tested a classifier that distinguished between MPX cases and healthy controls within our data set to obtain a feature importance (Fig. 3d). The most important features of the classifier (e.g., SERPINA3, AFM, PGLYRP2, and TF) overlapped with the differentially concentrated proteins in the COVID-19 plasma samples. Hence, within the limitations that our study is based on a small case series, our data suggests that there is sufficient overlap among host response proteins among different viral infections, that upon the disease specific adaptation of the statistical models, biomarker panel assay could be repurposed between different viral infections, for instance, to improve pandemic preparedness.*"

Minor issues:

1) page 2: line 12: COVID19 --> COVID-19

We have added the hyphen accordingly.

2) page 7, around line 15: please clarify the threshold of adjusted p values and fold-change for determining significantly regulated proteins for all the analyses of identification of differentially expressed proteins.

We extended the description of thresholds for finding differentially expressed proteins:

"*For finding regulated features, the following criteria were applied for all contrasts: Significance level alpha was set to 0.015, which guaranteed the Benjamini–Hochberg [37] false discovery rate below 5% for contrast MPX vs Control, below 4% for contrast COVID-19 vs Control, and below 22% for contrast MPX vs COVI-19. The log fold-change threshold was applied to all contrasts to guarantee that the measured signal is above the average noise level. As such we took the median residual standard deviation of linear model:* $log_2(T)$ *= median residual SD of linear modeling (=* $log_2(1.35)$*).*"

3) Fig 1c: please explain why the p.adjust was set as 0.3

In Figure 1c we showed enrichment of all terms, labeled are only terms below a certain threshold. The exact value of this threshold is of no scientific importance rather it is for illustration purposes only; we applied it in order to declutter the term labels in the lower part of the figure. The header of the figure seems to be confusing, and we have replaced it by

"GSEA of REACTOME terms".

4) Fig 1d, Fig2e, Fig 3c: please add p values for these box plots.

We thank the reviewer for the suggestion. The p-values for proteins in box plots Fig 1d were illustrated in Fig 1b. Nevertheless, following your advice we decided to add p-values and FDR in the plot and extend the figure caption for each protein:

"d) *Boxplots illustrating key proteins that differ between patients with MPX and controls (p-values and pfdr for corresponding contrast MPX vs Control are provided in brackets): TTR (p-value = 9E-10, fdr = 2E-7), LBP (p-value = 2E-4, fdr = 2E-3), APOC1 (p-value = 5E-8, fdr = 3E-6), and C9 (p-value = 2E-8, fdr = 2E-6)"*

The p-values for proteins in box-plots Fig2e is included in Fig 2d, but following the reviewer's advise we extended the figure caption by adding p-values and fdr for each protein:

"e) *Boxplots illustrating key proteins that differ between patients with MPX and COVID-19 (p-values and fdr for corresponding contrast MPX vs COVID-19 are provided in brackets): LCP1 (p-value = 3E-4, fdr = 3E-2), LDHB (p-value = 2E-3, fdr = 6E-2), CFHR1 (p-value = 2E-4, fdr = 3E-2)."*

For Fig 3c, we present p-values in TableEV 2, and we have added the respective p-values to the boxplot.

5) fig2d: are p valued adjusted?

P values are not adjusted in Fig 2d because we provided p-values from LIMMA moderated t-statistics. The chosen significance level for p-values (alpha = 0.015) ensured that for the contrast MPX vs COVID-19, fdr was below 22%. This FDR level was considered acceptable due to high similarity in proteome responses and the very small number of MPX samples in the study. We updated the figure caption. It now reads:

"**d)** *Volcano plot showing differentially regulated proteins of patients with MPX and COVID-19; $\alpha <= 0.015$ and $|logFC| >= 1.35$ were used for selection of regulated proteins. The chosen significance level ensured that fdr for this contrast was below 22%. "*

Referee #3 (Comments on Novelty/Model System for Author):

The model system is comprised of 5 patients diagnosed with Monkey Pox. Two of these patients also have HIV infections (though managed with minimal viral load). The authors have quantified 200-300 proteins in serum from these individuals and contrasted them against CoVID19 and age/gender matched non-infected/healthy controls. While they do observe significantly different features between the three datasets, the sample size and limited demographic range limit the power of this dataset to make strong conclusions. They also do some limited predictive modelling to support their observations which off a dataset of n=5, is not possible to judge the validity and should be removed.

We thank the Reviewer for engaging with our manuscript. While we agree with the Reviewer’s assessment that our case series study is of limited size i.e. for predictive modeling, we would like to highlight that the classifier has value for at other reasons: Please also refer to our replies to Reviewer #1 and #2, that made the same point. However, despite the moderate sample size, we believe our study is highly valuable, because it captures a clear proteomic response to a rapidly emerging infection of global importance, for which data is urgently needed. We hope our results help as a justification for conducting larger studies.

Referee #3 (Remarks for Author):

This manuscript is from a highly proficient and established group performing blood-proteomic analysis on clinical samples. The work here shows changes to proteins that arise during infection from Monkey Pox Virus in contrast to COVID 19 and healthy controls. The laboratory has a strong-established platform and track record for this type of analysis. Investigations into Monkey Pox infections are incredibly relevant given the current ongoing pandemic across Africa. As acknowledged by the authors, the principal limitation is the sample size (n=5) which is extremely small and does not represent a wide demographic, especially in relation to African populations. The authors perform some predictive modelling, though the purpose of this is not clear - with n=5 it is very limited to what conclusions can be taken from these, and it may be sensible to remove them altogether.

We thank the Reviewer for the positive assessment of the quality of our previous work. We fully agree with the limitations that arise from the small cohort size and have clearly stated them in the manuscript and in this response to the reviewers. Please see the comments above, specifically Reviewer #2 comment #1, explaining the rationale for constructing a machine learning classifier despite the moderate sample size of the case series study. In light of the reviewers’ valid comments, we have revised the study accordingly. Like the reviewer, we want to make sure that we do not oversell our results, but we would also like to emphasize that that study is biologically and medically highly valuable due to the timely importance to generate understanding about the current MPX outbreak. We believe a key value of our initial and timely characterisation of the MPX host response in the current outbreak, acquiring very clear signals, catalyzes the planning and conducting of larger and longitudinal studies.

That said, the authors also make valid discussion on the re-purposing of a biomarker test, using one developed by the same group for stratifying COVID19 patients and demonstrated

that it can also detect MPX infection. However, this raises further questions/concerns about the specificity of the tests as the key predictive proteins were the same.

We appreciate that there has been a drive for many years to find ‘specific’ biomarkers; but for the host response at least to a new viral agent this has always been an unlikely scenario from the biological point of view. That said, our data indicates that there is specificity: not in the identity of the markers themselves, but in the response patterns captured with modern and quantitative proteomic technologies. The discussion has been amended accordingly, to highlight the comment of the Reviewer.

“Second, our data suggested that there could be an untapped potential in the repurposing of biomarker panel assays across viral disease: although the overall proteomic signature clearly distinguished MPX from COVID19, there was a sufficient overlap in the host response signature, that we could distinguish MPX patients from healthy controls on the basis of a COVID19 proteomic panel assay. Indeed, despite our data hence suggests that the individual biomarkers are not specific to a particular infection, our analysis shows that the pattern in which they respond, seems highly discriminatory. Although these results are to be regarded preliminary due to the moderate the size of our cohort, our data suggests that one could generate a proteomic panel assay that is applicable across multiple viral disease; in case of a new viral agent, one could hence measure the same panel of biomarkers, and only would need to adapt the the data analysis and ML models to the novel agent. Future studies are needed to substantiate the viability of this possibility.”

Given the current MPX epidemic, this analysis is important, however for publication in a molecular-medical journal, a greater number and relevant more relevant demographic (the disease is prevalent in Africa, this cohort are (presumably) European as they were hospitalised in Germany?) need to be included.

We are currently working on extending our cohort, including additional investigations in Central Africa. However, the current outbreak mainly concerns MSM individuals in Europe and the Americas, such as the patients included in our case series. While we clearly acknowledge the need of expanding investigations to benefit previously endemic regions in Africa (which is clearly stated in the introduction and discussion section), we have deemed it appropriate and necessary, given the rapidly evolving outbreak, to conduct and to communicate the presented research without having collected a fully balanced and complete large cohort.

Aside from these major considerations, several minor points are raised below that should be addressed. Reviewed by Harry Whitwell, Imperial College London.

MINOR

1. The first sentence is missing comma's:

"The outbreak of Monkey pox (MPX), with more than 15,000 confirmed infections worldwide, is exception...."

We have changed the sentence accordingly.

2. I think the last sentence in the first paragraph should be:

"with generally higher case fatality associated with infections from the Central African viral clade compared to the West African virus clade [4]."

We have changed the sentence accordingly.

3. The second sentence in 3rd paragraph - "application" should be "applications".

We have changed the word accordingly.

4. In methods - you allow peptides with up to 40% missing values; this seems like a lot and suggests a possible problem with the MS acquisition. Can you justify this high level of inclusion and suggest what caused the missing values? This should extend to a justification of the choice of imputation method.

In plasma proteomics, one unfortunately deals with a higher number of missing values compared to the values one uses from cellular proteomics. There are two typical explanations given: plasma proteins have a high degree of modification. Second, trypsin is less effective on the highly compact plasma proteins, resulting in a higher number of missing cleavages. Finally, the huge dynamic range of the plasma proteome results in stronger thresholding effects. Addressing the reason for this is out of scope for this report focussing on the MPX proteome. To address the reviewer's comment, we have added a short comment about the nature of plasma proteomics and how we dealt with missing values into the Methods section:

"To deal with a higher number of missing values in plasma proteomics compared to those obtained from cellular proteomics, we adopted the following approach. Peptides with excessive missing values (> 40 % per group) were excluded from our analysis. This group-based thresholding delivered approximately the same number of peptides as the 26 % presence threshold applied to the total set. The missing values of remaining peptides were imputed group-based using the PCA method (Josse & Husson, 2016). The group-based imputation allowed to avoid admixing of information from other groups."

In methods - can you justify the rational for the protein expression transformation: log2(1+Nlesions-/15)?

Log base 2 transformation $f(N) = \log2(1+N/N_{mean})$ was applied to bring the number of lesions to the same scale as protein expressions, 1 was added to guarantee that N lesions = 0 is transformed to 0 and division by N_{mean}=15 (average number of lesions) was applied to map

the average lesions number to 1. Please also note that for 0 < N/Na <1.5 deviations of f(N) from linearity are less than 12%. We have added a rationale to the Methods section:

"Log base 2 transformation was applied to bring the number of lesions to the same scale as protein expressions, 1 was added to guarantee that N lesions = 0 is transformed to 0 and division by Nmean=15 (average number of lesions) was applied to map the average lesions number to 1. Also note that for 0 < N/Nmean <1.5 deviations of f(N) from linearity are less than 12%."

5. In methods (first paragraph in Discovery proteomics data analysis), I cannot find a reference to PLM in reference 35. And the Bioconductor page for the package does not contain a description either. Please give the full name and a suitable reference to describe what this is doing to derive protein-level expression data from the peptide values.

We thank the reviewer for the comment on reference. The reference of PLM is in the referred book, in index "PLM, see Probe level Model (PLM)" → pages 46 – 47. We provided this reference because Chapter 3 is written by B. Bolstad, developer of the PLM method, and provides a very detailed explanation of the method, the math behind and comparison with Tukey's median polish. We have updated the reference by adding the chapter name and pages:

"Bolstad B (2008) Preprocessing and Normalization for Affymetrix GeneChip Expression repoMicroarrays. In Methods in Microarray Normalization, Stafford P (ed) pp 41–59. Taylor & Francis"

6. Table 1 - two ranges are given in the third column - what are these? Matched data for controls should be provided in the same table in the main text.

Due to the cohort size we decided to provide IQR and range, both in the right column. We have clarified the table legend and labeled the data in the column. Since the controls are matched on basic demographic data (age, gender) and most of the data in table 1 is MPX cohort specific we decided to move the controls to another table. However, in order to facilitate a comparison at first glance we have duplicated the basic demographic data (age, gender, BMI) for all three cohorts in TableEV 3.

7. How were the MPX patients treated, was a sample taken before any hospital intervention?

None of the patients received MPX specific treatment. As severe (peri-) anal pain was the chief complaint, all patients immediately received intravenous and oral analgesiacs at presentation, thus no pre-"treatment" samples are available.

8. Typo P11, line 23 "thia" should be "this".

We have changed the typo.

9. How many quantitative points over each peptide LC peak? - this should be reported as you are aiming to "[obtain] maximally precise proteomics measurements". The CV values presented in the results (25% and 34%) - are these median CV across all peptides? How many peptides achieved this CV? The yaxis label for Sup Fig 1C is missing. How successful was the normalisations? The iRT was spiked into each sample, so could be compared across samples (should have minimal CV calculated within each batch and across all batches). Otherwise, it is not clear what the purpose of adding these was.

At a duty cycle of 1.4s, we measure on average 7 points per chromatographic peak. This information has been included in the manuscript.

"A Zeno SWATH acquisition scheme with 85 variable-size windows and 11-ms accumulation time with 1.4s cycle time was used (Wang et al, 2022) allows for MS detection for average 7 points per chromatographic peak with the chosen chromatography."

Presented median CVs are across the 226 consistently quantified proteins. Median means that half of the proteins have CV below provided values. Success of normalization and preprocessing in general can be seen from QC charts (Supplementary Figure 1a and 1b), showing that CV of the median of the protein distribution is ~1.4% and CV for IQR is ~ 4.2%, indicating very good alignment of protein distributions across all samples. Note that normalization, as described in the Methods and Materials section, was carried out at the precursor level.

10. Fig2B axis should say log-FC to help with interpretation. Assuming that "!" mean "not" in the top legend - this nomenclature should be explained in the legend. In fact, the legends here are confusing and it took a while for me to get it. The two legends need to be next to each other to help the reader make sense of it. I would advise re-thinking the legend altogether to describe what each point means. I think there is probably space in the top left and bottom right corner of the plot to give a description of each point like how it is given in the legend. Especially as the figure is large (1page) so the legend may not be on the same page as the figure.

We thank the reviewer for the comment on figure legends. We have changed Fig 2 and legends were provided in a concise manner, and a detailed explanation of each group is provided in the figure caption for enhanced clarity. Following the reviewer's advice we added logFC to the axis labels, moved the first legend into the top left corner, expanded the second legend, and placed it in the header:

MPX − Control as Contrast1 (C1)

COVID-19 − Control as Contrast2 (C2)

MPX − COVID-19 as Contrast3 (C2)

Reg: regulated, !: not

11. In results: "Furthermore, we found 19 proteins that were differentially abundant in MPX but not in COVID-19. For instance, LCP1 and LDHB were found to be only upregulated in MPX (Fig 2 d, e)" - fold change and p-value should be given to help with interpretation. The

sample size is only small, therefore the power to detect small changes is probably very limited.

We completely agree with the reviewer that with a higher sample number, we would have most likely been able to identify more of the smaller changes, therefore we added one sentence for explanation. Nonetheless, the proteins we reported were consistently differentially expressed in MPX and are statistically significant, and hence worth reporting. Indeed, many of the proteins identified in this section have a known role in the antiviral response, lending additional confidence in our results (see discussion). To address the reviewer's point, we have added a better explanation to the manuscript regarding the limitations of our study in this respect, and which benefits larger studies will bring in the future,.

Results 'Of note, it is plausible that additional differentially abundant proteins can be identified by proteomics in other or larger cohorts.'

Discussion: "*Indeed, we hope that the clear proteomic signature revealed by our case series justifies larger studies involving different cohorts and longitudinal samples in the near future.*"

12. Results - the classifier between for MPX vs Control and MPX vs CoVID contains 8 proteins (fig 2f) (though 5 proteins are mentioned in the methods?) and is built based on 5 samples..... I'm not sure this adds anything - the sample size is limited (as acknowledged), in my view, this analysis does nothing to support the other observations. I would drop it.

Please see our reply to Reviewer #2 comment #1. The main intention of including the cross-validated ML analysis is to provide an orthogonal analysis of the MPX vs COVID-19 and healthy control proteomes, and to back up the DE analysis with a feature ranking exercise. We hope our revisions have satisfactorily addressed the rationale. Please see our reply to Reviewer #2.

13. I found the re-purposing of biomarker panels provocative and certainly worthy of inclusion for support of this important concept. The generation of a predictive model based on only 5 cases again is likely overfitted - but the point is to show that this panel may be repurposed rather than to propose a test directly. Therefore, I think as presented it is of interest. Of concern though is the specificity to differentiate between the diseases. In general, the fold-change difference between differentiating proteins is not great and could be due to chance. This needs further discussion to be convincing. SERPINA3 and APOA1 were both similarly changing in the discovery proteomics dataset for both MPX and COVID19 vs controls (red dots in Fig2b). Therefore, would this classifier be expected to differentiate between diseases? - probably not (you also show this in the classifier figures).

This comment overlaps with the point the Reviewer made in his summary. The ML model is not overfitted but we agree with the above-mentioned constraint of the cohort size. Indeed however, we did not expect human biomarkers to be fully specific to a single viral infection, but it is encouraging to see that the panels/patterns are highly discriminatory against healthy controls, at least in our cohort. Nonetheless, we agree with both reviewers that the point about panel repurposing, although plausible, is to be seen as speculative at the moment due

to the small cohort size, and have toned down the part accordingly.

14. I think the last sentence in the results is missing something - I'm not following its meaning (perhaps should be "...in the `COVID19` disease plasma"?).

We have now rephrased the whole paragraph:

"*Based on this proteomics data, we tested a classifier that distinguished between MPX cases and healthy controls within our data set to obtain a feature importance (Fig. 3d). The most important features of the classifier (e.g., SERPINA3, AFM, PGLYRP2, and TF) overlapped with the differentially concentrated proteins in the COVID-19 plasma samples. Hence, within the limitations that our study is based on a small case series, our data suggests that there is sufficient overlap among host response proteins among different viral, that upon the disease specific adaptation of the statistical models, biomarker panel assay could be repurposed between different viral infections, for instance, to improve pandemic preparedness*."

8th Sep 2022

Thank you for the submission of your revised manuscript to EMBO Molecular Medicine. We have now received the enclosed report from the three referees who were asked to re-assess it. As you will see, the referees are now supportive, and I am pleased to inform you that we will be able to accept your manuscript pending the following amendments:

In the event of acceptance, this file will be published in conjunction with your paper and will include the anonymous referee reports, your point-by-point response, and all pertinent correspondence relating to the manuscript. Let us know whether you DISAGREE with this and if you want to remove or keep any figures from it prior to publication.

Please note that the Authors checklist will be published at the end of the RPF.

Please submit your revised manuscript within two weeks. I look forward to seeing a revised form of your manuscript as soon as possible.

Kind regards,
Jingyi

Jingyi Hou
Editor
EMBO Molecular Medicine

***** Reviewer's comments *****

Referee #1 (Comments on Novelty/Model System for Author):

All queries have been adequately addressed.

Referee #1 (Remarks for Author):

-

Referee #2 (Comments on Novelty/Model System for Author):

The sample size is limited. Lack of independent validation or mechanistic study.

Referee #2 (Remarks for Author):

Thanks for the revision. I have no more question.

Referee #3 (Comments on Novelty/Model System for Author):

The model system is small but very timely. The data is of importance due to the current emergence of MPX.

Referee #3 (Remarks for Author):

I am happy that all my points have been addressed by the authors. While the cohort is small, the manuscript is timely and of current interest.

The authors performed the requested editorial changes.

9th Sep 2022

We are pleased to inform you that your manuscript is accepted for publication and is now being sent to our publisher to be included in the next available issue of EMBO Molecular Medicine.

We would like to remind you that as part of the EMBO Publications transparent editorial process initiative, EMBO Molecular Medicine will publish a Review Process File online to accompany accepted manuscripts. If you do NOT want the file to be published or would like to exclude figures, please immediately inform the editorial office via e-mail.

Please read below for additional IMPORTANT information regarding your article, its publication and the production process.

Thank you again for sending this interesting work to EMBO Mol Med!

Best wishes,
Jingyi

Jingyi Hou
Editor
EMBO Molecular Medicine

Follow us on Twitter @EmboMolMed
Sign up for eTOCs at embopress.org/alertsfeeds

EMBO Press Author Checklist

Corresponding Author Name: Markus Ralser	
Journal Submitted to: EMBO Molecular Medicine	
Manuscript Number: EMM-2022-16643	

USEFUL LINKS FOR COMPLETING THIS FORM
The EMBO Journal - Author Guidelines
EMBO Reports - Author Guidelines
Molecular Systems Biology - Author Guidelines
EMBO Molecular Medicine - Author Guidelines

Reporting Checklist for Life Science Articles (updated January 2022)
This checklist is adapted from Materials Design Analysis Reporting (MDAR) Checklist for Authors. MDAR establishes a minimum set of requirements in transparent reporting in the life sciences (see Statement of Task: 10.31222/osf.io/9sm4x). Please follow the journal's guidelines in preparing your manuscript.
Please note that a copy of this checklist will be published alongside your article.

Abridged guidelines for figures
1. Data
The data shown in figures should satisfy the following conditions:
- the data were obtained and processed according to the field's best practice and are presented to reflect the results of the experiments in an accurate and unbiased manner.
- ideally, figure panels should include only measurements that are directly comparable to each other and obtained with the same assay.
- plots include clearly labeled error bars for independent experiments and sample sizes. Unless justified, error bars should not be shown for technical replicates.
- if n<5, the individual data points from each experiment should be plotted. Any statistical test employed should be justified.
- Source Data should be included to report the data underlying figures according to the guidelines set out in the authorship guidelines on Data Presentation.

2. Captions
Each figure caption should contain the following information, for each panel where they are relevant:
- a specification of the experimental system investigated (eg cell line, species name).
- the assay(s) and method(s) used to carry out the reported observations and measurements.
- an explicit mention of the biological and chemical entity(ies) that are being measured.
- an explicit mention of the biological and chemical entity(ies) that are altered/varied/perturbed in a controlled manner.
- the exact sample size (n) for each experimental group/condition, given as a number, not a range;
- a description of the sample collection allowing the reader to understand whether the samples represent technical or biological replicates (including how many animals, litters, cultures, etc.).
- a statement of how many times the experiment shown was independently replicated in the laboratory.
- definitions of statistical methods and measures:

- common tests, such as t-test (please specify whether paired vs. unpaired), simple χ2 tests, Wilcoxon and Mann-Whitney tests, can be unambiguously identified by name only, but more complex techniques should be described in the methods section;

- are tests one-sided or two-sided?
- are there adjustments for multiple comparisons?
- exact statistical test results, e.g., P values = x but not P values < x;
- definition of 'center values' as median or average;
- definition of error bars as s.d. or s.e.m.

Please complete ALL of the questions below.
Select "Not Applicable" only when the requested information is not relevant for your study.

Materials

Newly Created Materials	Information included in the manuscript?	In which section is the information available? (Reagents and Tools Table, Materials and Methods, Figures, Data Availability Section)
New materials and reagents need to be available; do any restrictions apply?	Not Applicable	

Antibodies	Information included in the manuscript?	In which section is the information available? (Reagents and Tools Table, Materials and Methods, Figures, Data Availability Section)
For **antibodies** provide the following information:<br>- Commercial antibodies: RRID (if possible) or supplier name, catalogue number and or/clone number<br>- Non-commercial: RRID or citation	Not Applicable	

DNA and RNA sequences	Information included in the manuscript?	In which section is the information available? (Reagents and Tools Table, Materials and Methods, Figures, Data Availability Section)
**Short novel DNA or RNA including primers, probes**: provide the sequences.	Yes	The partial sequence of the genome of the MPXV isolate obtained from one of the patients was determined and is available on GenBank (ON813251.2).

Cell materials	Information included in the manuscript?	In which section is the information available? (Reagents and Tools Table, Materials and Methods, Figures, Data Availability Section)
**Cell lines:** Provide species information, strain. Provide accession number in repository **OR** supplier name, catalog number, clone number, and/**OR** RRID.	Not Applicable	
**Primary cultures:** Provide species, strain, sex of origin, genetic modification status.	Not Applicable	
Report if the cell lines were recently **authenticated** (e.g., by STR profiling) and tested for mycoplasma contamination.	Not Applicable	

Experimental animals	Information included in the manuscript?	In which section is the information available? (Reagents and Tools Table, Materials and Methods, Figures, Data Availability Section)
**Laboratory animals or Model organisms:** Provide species, strain, sex, age, genetic modification status. Provide accession number in repository **OR** supplier name, catalog number, clone number, **OR** RRID.	Not Applicable	
**Animal observed in or captured from the field:** Provide species, sex, and age where possible.	Not Applicable	
Please detail **housing and husbandry conditions**.	Not Applicable	

Plants and microbes	Information included in the manuscript?	In which section is the information available? (Reagents and Tools Table, Materials and Methods, Figures, Data Availability Section)
**Plants:** provide species and strain, ecotype and cultivar where relevant, unique accession number if available, and source (including location for collected wild specimens).	Not Applicable	
**Microbes:** provide species and strain, unique accession number if available, and source.	Not Applicable	

Human research participants	Information included in the manuscript?	In which section is the information available? (Reagents and Tools Table, Materials and Methods, Figures, Data Availability Section)
If collected and within the bounds of privacy constraints report on age, sex and gender or ethnicity for all study participants.	Yes	Materials and Methods, Data Availablility section, Supplementary Data

Core facilities	Information included in the manuscript?	In which section is the information available? (Reagents and Tools Table, Materials and Methods, Figures, Data Availability Section)
If your work benefited from core facilities, was their service mentioned in the acknowledgments section?	Yes	Core facility members co-author the manuscipt

Design

Study protocol	Information included in the manuscript?	In which section is the information available? (Reagents and Tools Table, Materials and Methods, Figures, Data Availability Section)
If study protocol has been **pre-registered, provide DOI in the manuscript**. For clinical trials, provide the trial registration number **OR** cite DOI.	Not Applicable	
Report the **clinical trial registration number** (at ClinicalTrials.gov or equivalent), where applicable.	Not Applicable	

Laboratory protocol	Information included in the manuscript?	In which section is the information available? (Reagents and Tools Table, Materials and Methods, Figures, Data Availability Section)
Provide DOI OR other citation details if **external detailed step-by-step protocols** are available.	Not Applicable	

Experimental study design and statistics	Information included in the manuscript?	In which section is the information available? (Reagents and Tools Table, Materials and Methods, Figures, Data Availability Section)
Include a statement about **sample size** estimate even if no statistical methods were used.	Yes	All sample sizes are clearly stated throughout the manuscript
Were any steps taken to minimize the effects of subjective bias when allocating animals/samples to treatment (e.g. **randomization procedure**)? If yes, have they been described?	Yes	We have taken several precautions to avoid batch effcts in our proteomic method. Methods section
Include a statement about **blinding** even if no blinding was done.	Yes	Blinding statement included
Describe **inclusion/exclusion criteria** if samples or animals were excluded from the analysis. Were the criteria pre-established?<br><br>If sample or data points were omitted from analysis, report if this was due to attrition or intentional exclusion and provide justification.	Yes	Inclusion/exclusion criteria are detailed in the matierals methods section. Peptides with excessive missing values were excluded from the analysis as described in the Materials and Methods section
For every figure, are **statistical tests** justified as appropriate? Do the data meet the assumptions of the tests (e.g., normal distribution)? Describe any methods used to assess it. Is there an estimate of variation within each group of data? Is the variance similar between the groups that are being statistically compared?	Yes	Materials and Methods section describes statistical methods applied. Values for within group coefficient of variation are provided in the Results section.

Sample definition and in-laboratory replication	Information included in the manuscript?	In which section is the information available? (Reagents and Tools Table, Materials and Methods, Figures, Data Availability Section)
In the figure legends: state number of times the experiment was **replicated** in laboratory.	Yes	Information about replication is provied in Figure legends and MM
In the figure legends: define whether data describe **technical or biological replicates**.	Yes	Information about replication is provied in Figure legends and MM

Ethics

Ethics	Information included in the manuscript?	In which section is the information available? (Reagents and Tools Table, Materials and Methods, Figures, Data Availability Section)
Studies involving **human participants**: State details of **authority granting ethics approval** (IRB or equivalent committee(s), provide reference number for approval.	Yes	Materials and Methods
Studies involving **human participants**: Include a statement confirming that **informed consent** was obtained from all subjects and that the experiments conformed to the principles set out in the WMA Declaration of Helsinki and the Department of Health and Human Services Belmont Report.	Yes	Materials and Methods
Studies involving **human participants:** For publication of **patient photos**, include a statement confirming that consent to publish was obtained.	Not Applicable	
Studies involving experimental **animals**: State details of **authority granting ethics approval** (IRB or equivalent committee(s), provide reference number for approval. Include a statement of compliance with ethical regulations.	Not Applicable	
Studies involving **specimen and field samples:** State if relevant **permits** obtained, provide details of authority approving study; if none were required, explain why.	Not Applicable	

Dual Use Research of Concern (DURC)	Information included in the manuscript?	In which section is the information available? (Reagents and Tools Table, Materials and Methods, Figures, Data Availability Section)
Could your study fall under dual use research restrictions? Please check biosecurity documents and list of **select agents and toxins** (CDC): https://www.selectagents.gov/sat/list.htm	Not Applicable	
If you used a select agent, is the security level of the lab appropriate and reported in the manuscript?	Not Applicable	
If a study is subject to dual use research of concern regulations, is the name of the **authority granting approval and reference number** for the regulatory approval provided in the manuscript?	Not Applicable	

Reporting

The MDAR framework recommends adoption of discipline-specific guidelines, established and endorsed through community initiatives. Journals have their own policy about requiring specific guidelines and recommendations to complement MDAR.

Adherence to community standards	Information included in the manuscript?	In which section is the information available? (Reagents and Tools Table, Materials and Methods, Figures, Data Availability Section)
State if relevant guidelines or checklists (e.g., **ICMJE, MIBBI, ARRIVE, PRISMA**) have been followed or provided.	Not Applicable	
For **tumor marker prognostic studies,** we recommend that you follow the **REMARK** reporting guidelines (see link list at top right). See author guidelines, under 'Reporting Guidelines'. Please confirm you have followed these guidelines.	Not Applicable	
For **phase II and III randomized controlled trials**, please refer to the **CONSORT** flow diagram (see link list at top right) and submit the CONSORT checklist (see link list at top right) with your submission. See author guidelines, under 'Reporting Guidelines'. Please confirm you have submitted this list.	Not Applicable	

Data Availability

Data availability	Information included in the manuscript?	In which section is the information available? (Reagents and Tools Table, Materials and Methods, Figures, Data Availability Section)
Have **primary datasets** been deposited according to the journal's guidelines (see 'Data Deposition' section) and the respective accession numbers provided in the Data Availability Section?	Yes	Data Availability
Were **human clinical and genomic datasets** deposited in a public access-controlled repository in accordance to ethical obligations to the patients and to the applicable consent agreement?	Yes	Key clinical data as well as proteomic data are published alongside the manuscript as TableEV 1 and the Pride repository. The manusccript contains no sensitive data that needs access control.
Are **computational models** that are central and integral to a study available without restrictions in a machine-readable form? Were the relevant accession numbers or links provided?	Not Applicable	
If publicly available data were reused, provide the respective **data citations in the reference list**.	Not Applicable	
